# Analysis of *Potato virus Y* Coat Protein Epitopes Recognized by Three Commercial Monoclonal Antibodies

**DOI:** 10.1371/journal.pone.0115766

**Published:** 2014-12-26

**Authors:** Yan-Ping Tian, Jussi Hepojoki, Harri Ranki, Hilkka Lankinen, Jari P. T. Valkonen

**Affiliations:** 1 Department of Agricultural Sciences, Plant Pathology Laboratory, University of Helsinki, Helsinki, Finland; 2 Department of Virology, Peptide and Protein Laboratory, Infection Biology Research Program, Haartman Institute, University of Helsinki, Helsinki, Finland; Washington State University, United States of America

## Abstract

**Background:**

*Potato virus Y* (PVY, genus *Potyvirus*) causes substantial economic losses in solanaceous plants. Routine screening for PVY is an essential part of seed potato certification, and serological assays are often used. The commercial, commonly used monoclonal antibodies, MAb1128, MAb1129, and MAb1130, recognize the viral coat protein (CP) of PVY and distinguish PVY^N^ strains from PVY^O^ and PVY^C^ strains, or detect all PVY strains, respectively. However, the minimal epitopes recognized by these antibodies have not been identified.

**Methodology/Principal Findings:**

SPOT peptide array was used to map the epitopes in CP recognized by MAb1128, MAb1129, and MAb1130. Then alanine replacement as well as N- and C-terminal deletion analysis of the identified peptide epitopes was done to determine critical amino acids for antibody recognition and the respective minimal epitopes. The epitopes of all antibodies were located within the 30 N-terminal-most residues. The minimal epitope of MAb1128 was ^25^NLNKEK^30^. Replacement of ^25^N or ^27^N with alanine weakened the recognition by MAb1128, and replacement of ^26^L, ^29^E, or ^30^K nearly precluded recognition. The minimal epitope for MAb1129 was ^16^RPEQGSIQSNP^26^ and the most critical residues for recognition were ^22^I and ^23^Q. The epitope of MAb1130 was defined by residues ^5^IDAGGS^10^. Mutation of residue ^6^D abrogated and mutation of ^9^G strongly reduced recognition of the peptide by MAb1130. Amino acid sequence alignment demonstrated that these epitopes are relatively conserved among PVY strains. Finally, recombinant CPs were produced to demonstrate that mutations in the variable positions of the epitope regions can affect detection with the MAbs.

**Conclusions/Significance:**

The epitope data acquired can be compared with data on PVY CP-encoding sequences produced by laboratories worldwide and utilized to monitor how widely the new variants of PVY can be detected with current seed potato certification schemes or during the inspection of imported seed potatoes as conducted with these MAbs.

## Introduction

*Potato virus Y* (PVY) is the type member of the genus *Potyvirus* (family *Potyviridae*), the largest genus of plant-infecting RNA viruses [1]. PVY has a positive-sense single-stranded RNA genome (∼9700 nt, depending on the strain) containing a single open reading frame that encodes a large polyprotein cleaved into ten mature proteins by three virally encoded proteinases. An additional open reading frame (*pipo*, 75 codons) is created by frame-shifting in the region encoding protein P3 [2]. PVY genome is encapsidated to filamentous particles comprised of the viral CP (267 amino acid residues) that is the most C′-terminal region of the polyprotein and released by the viral proteinase NIa [1].

PVY mainly infects plants of family Solanaceae and causes great yield losses in major crops, including potato (*Solanum tuberosum* L.) [3], tobacco (*Nicotiana tabacum* L.) [4] and pepper (*Capsicum* spp.) [5]. Aphids transmit PVY nonpersistently in the field, and a short visit and probing of the plant by an aphid is sufficient for transmission. The use of insecticides is insufficient to prevent PVY spread, whereas spraying foliage with mineral oil to interfere with probing, and use of straw mulch to reduce the contrast between plants and soil, can reduce PVY transmission [6].

The most common and important sources of PVY to potato crops are seed tubers infected with PVY [3] and the plants growing from them, as they offer a PVY source to aphids for further dissemination of the virus in the field. Therefore, planting virus-indexed healthy seed potatoes is an essential prerequisite to control potato yield losses caused by PVY. Special seed potato production and certification systems are needed to ensure the quality. Visual inspection of virus symptoms in the foliage of seed potato crops in the field is done to rogue the infected plants, but it is not a reliable or practical means to detect PVY in all potato cultivars, because PVY symptoms are not always characteristic enough, other symptoms may mask PVY symptoms, and some PVY strains cause no symptoms in certain cultivars (for symptoms caused by two PVY isolates in different cultivars grown from infected seed tubers, visit http://www.helsinki.fi/ppvir/research/pvy/index.html). Furthermore, current-season infections may cause no symptoms in foliage, although the progeny tubers will be infected. Therefore, seed potatoes need to be indexed for PVY using virus-specific, sensitive diagnostic methods.

The most efficient means to control PVY is a potato cultivar's native resistance to PVY [7]-[10]. Resistance genes recognizing and conferring high levels (extreme) resistance to all PVY strains exist but are relatively rare in potato cultivars [11]. Other resistance genes recognize only certain groups of PVY strains. They trigger a hypersensitive resistance response (HR) in potato and prevent PVY from spreading to other parts of the plants from the initial infection site. The HR genes *Ny_tbr_* and *Nc_tbr_* are common in potato cultivars [7]–[9]. The strains of PVY recognized by these genes are designated to strain groups PVY^O^ and PVY^C^, respectively [8], [12]. However, PVY strains not recognized by *Ny_tbr_* and *Nc_tbr_* have become prevalent in all potato production areas and are now the cause of major crop losses. These strains designated to strain group PVY^N^ [12] have been less of a concern for potato production in past, because they are often symptomless or cause only mild symptoms and limited yield reduction in potato [3]. However, the currently predominant PVY^N^ strains are recombinants [13]. They carry genomic segments of PVY^O^ strains and cause acute diseases in potato, including necrotic symptoms in tubers and leaves, and are called NTN strains within the PVY^N^ strain group. Therefore, it is important to detect PVY using antibodies recognizing specific strain groups, notably the PVY^N^, so to eliminate the seed lots carrying PVY strains that can overcome resistance in the locally grown potato cultivars.

Serological detection of PVY relies on detection of CP (virus particles) with polyclonal (PAb) or monoclonal antibodies (MAb) and is commonly carried out using the enzyme-linked immunosorbent assay (ELISA) [14], [15]. Additionally, polymerase chain reaction (PCR)-based methods that detect viral nucleic acids are often used [16], [17], but they tend to be more costly, require more advanced laboratory facilities than ELISA, and may still require antibodies for immunocapture, i.e., trapping and concentrating virions from plant sap [18]–[20]. Studies on *Potato virus A* (PVA, genus *Potyvirus*) indicate that, in potyvirus particles, CP folds such that the N-terminus is exposed at the surface of the particles [21]. Therefore, immunization of animals with potyvirus virions results in antibodies whose epitopes are mainly in the N-terminal region of CP [15].

Polyclonal antisera produced in animals immunized with PVY virions do not distinguish the PVY strain groups, but MAbs are needed [22]. Early studies on MAbs to PVY showed that immunization of mice with PVY^N^ strain 605 virions resulted in hybrid cell cultures producing antibodies specific to PVY^N^ strains that did not detect PVY^O^ or PVY^C^ strains. On the other hand, many MAbs detected all PVY strains indiscriminately [23], indicating that they detected epitopes highly conserved in PVY CP and could be used for general detection of PVY. Since then, MAbs have been prepared for PVY strain group specific detection or general detection of PVY strains in many research laboratories and companies [24]–[31], but the epitopes of only relatively few MAbs have been studied. Keller et al. [15] prepared a phage library expressing 12-amino acid long random peptides and used three MAbs produced for detection of PVY [25] to select peptides from the library. Alignment of the peptide sequences with the CP sequence of PVY^N^ suggested that each MAb detected a different epitope located within the N′-proximal part of the CP, which was confirmed by detection of the recombinant N-proximal part (55 residues) of the PVY^N^ CP with the MAbs. The residues of the epitopes crucial for detection were predicted but not verified experimentally. Comparison of the peptide sequences with the CP sequences of PVY^N^, PVY^N^-NTN, PVY^O^ and PVY^C^ suggested that one MAb was specific to PVY^N^ (including PVY^N^-NTN) and two MAbs detected all PVY strains, but these specificities were not tested. Nikolaeva et al. [31] mapped the binding sites of three commercially available PVY^N^-specific MAbs in the N′-proximal region of CP using overlapping synthetic peptides of different length.

A single amino acid substitution can abolish or create an epitope recognized by a MAb [32]. Chikh Ali et al. [33] concluded that a glycine residue in CP at position 29 allows detection of the PVY^N^ isolate PVY-12 with MAb2 that is supposed to detect PVY^O^ and PVY^C^ strains [26], and also allows detection of PVY-12 with MAb 1F5 that reacts mainly with PVY^N^ strains [28]. In contrast, PVY^N^-like isolates carrying a glutamic acid residue at position 29 are detected only with MAb 1F5 [33]. Karasev et al. [34] concluded that glutamine at position 98 of PVY CP is required for detection with MAb 1F5. Recently, surface plasmon resonance analysis was used successfully to test which anti-PVY CP MAbs share the same epitope, but the epitopes were not localized in CP [35].

There is little information about the minimal epitopes of any PVY-specific MAbs. Such information would be useful for predicting *in silico* cross-reactivity of antibodies [15] and which virus isolates may escape detection. Minimal epitopes can be determined using alanine replacement (alanine scanning) and/or N- and C-terminal deletion analyses of synthetic peptides, e.g., as reported with *Potato mop-top virus* (genus *Pomovirus*) [36] and *Potato virus A* (genus *Potyvirus*) [37]. Therefore, the aim of this study was to determine the minimal epitopes of three commercial MAbs that are commonly used to detect PVY in potato, especially during seed potato certification. According to the supplier of the MAbs (Neogen Phytodiagnostics, Ayr, Scotland), it is believed that MAb1128 detects PVY^N^ specifically and MAb1129 detects both PVY^O^ and PVY^C^ that are closely related [38], whereas MAb1130 detects all three strain groups of PVY. Hence, the three MAbs can be used to determine the overall incidence of PVY in seed potato lots or the specific incidence of PVY^N^ vs. PVY^O^ and PVY^C^. However, because the epitopes corresponding to the three MAbs in PVY CP are unknown, it is difficult to compare the molecular PVY strain data based on CP-encoding sequences or the detection by other MAbs and to predict how exclusively PVY isolates might be detected based on the three MAbs. Therefore, we determined the minimal epitopes recognized by the three MAbs using overlapping synthetic peptides, alanine scanning mutagenesis, peptide deletion analyses, and CP point mutants.

## Results

### Mapping Epitopes on CP Using SPOT Peptide Array

SPOT peptide synthesis [39] was used to generate 20 aa–long overlapping peptides at 3-aa intervals covering CP of the PVY^O^ strain SASA207 (GenBank accession no. AJ584851), PVY^N^ strain 605 (X97895), and PVY^C^ strain Adgen (AJ890348), and an additional potyvirus species, *Potato virus V* (PVV, genus *Potyvirus*, isolate Suomi, AJ278894). Each CP was therefore represented by 84 overlapping peptides on a modified cellulose membrane and probed individually with MAb1128, MAb1129, or MAb1130.

**Figure 1:**
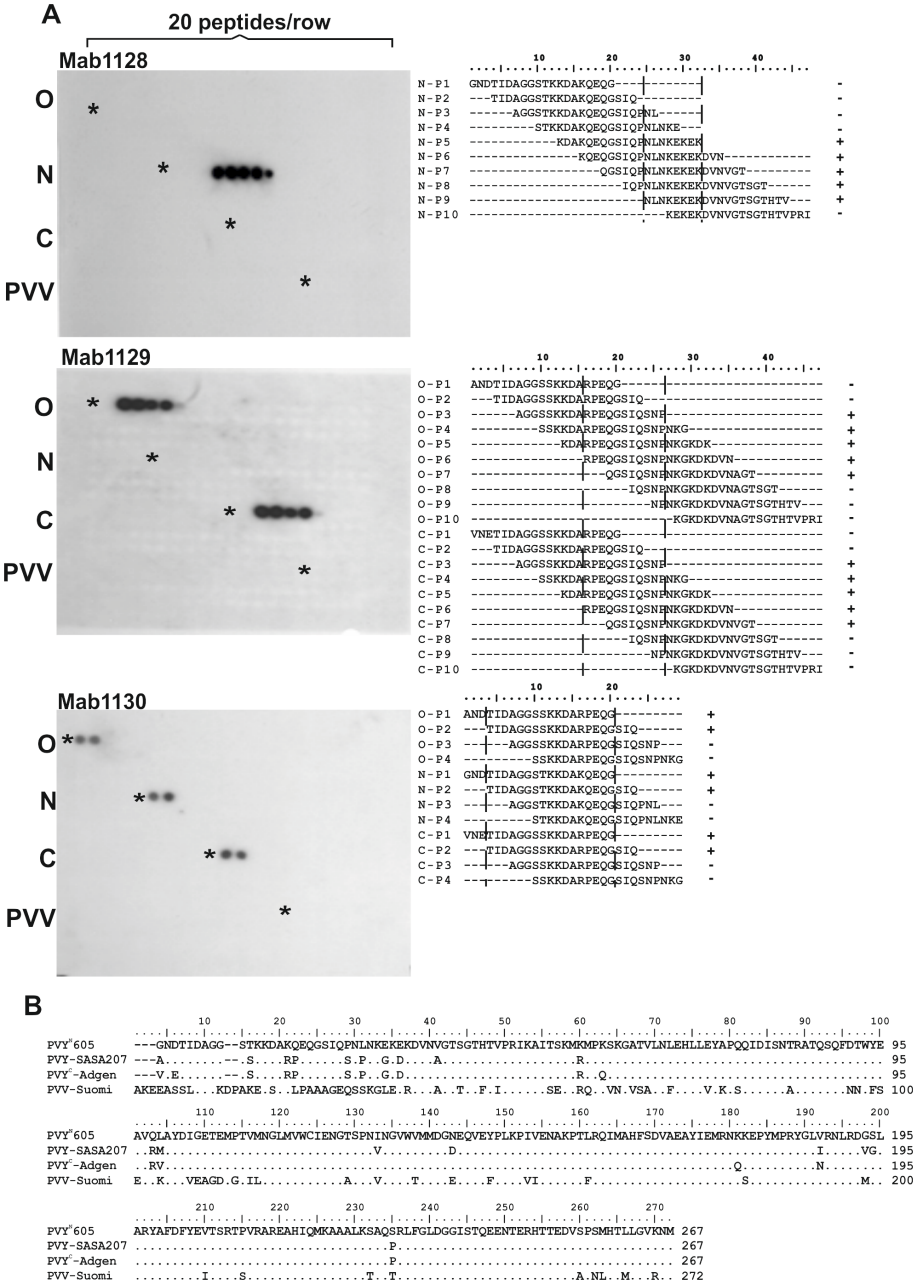
Mapping of the epitopes detected by monoclonal antibodies in three PVY strains. **A.** Each CP of *Potato virus Y* (PVY) strains O (PVY^O^-SASA207), N (PVY^N^-605) and C (PVY^C^-Adgen), and *Potato virus V* (PVV), was represented by 84 overlapping 20-residue long peptides synthesized on the membrane. Each row of the membrane contained 20 peptides. Asterisks indicate the position of the most N-terminal peptide of each virus. The membrane was probed with each of the following antibodies against CP: MAb1128, MAb1129, and MAb1130 (panels to the left). Binding of the MAbs to peptides was detected with horseradish peroxidase–labeled anti-mouse antibodies and visualized by capturing the enhanced chemiluminescence signals on X-ray film. The peptides detected with the respective MAb are aligned to the right (+, positive detection; –, negative). The sequence common to all peptides detected with each MAb is demarcated with dashed vertical lines. The positions of the amino acid residues in CP are indicated on top of the alignment. **B.** Complete amino acid sequences of PVY^N^-605, PVY^O^-SASA207, PVY^C^-Adgen, and PVV-Suomi aligned using Clustal X 2.0. Dots indicate identical residues, and dashes indicate gaps.

MAb1128 recognized five peptides in the N-terminal region of PVY^N^ CP, but it did not react with the peptides of other PVY strains (Fig. 1, top left). The sequences of the MAb1128-reactive peptides (Fig. 1, top right) share a common motif, ^25^NLNKEKEK^32^ (aa numbering based on complete PVY CP). MAb1129 detected five peptides in the N-terminal region of both PVY^O^ and PVY^C^ (Fig. 1, middle left), but not PVY^N^, and the peptides share a common motif, ^16^RPEQGSIQSNP^26^ (Fig. 1, middle right). MAb1130 reacted with two peptides of each of the three PVY strains (Fig. 1, bottom left), and these peptides correspond to the N-proximal portion of CPs. The common peptide sequence detected by MAb1130 in the three PVY strains was ^4^TIDAGGSSKKDARPEQG^20^, but there were two aa substitutions in PVY^N^ (S11T and R16K; Fig. 1, bottom right). PVV peptides were not recognized by any of the MAbs (Fig. 1).

**Figure 2:**
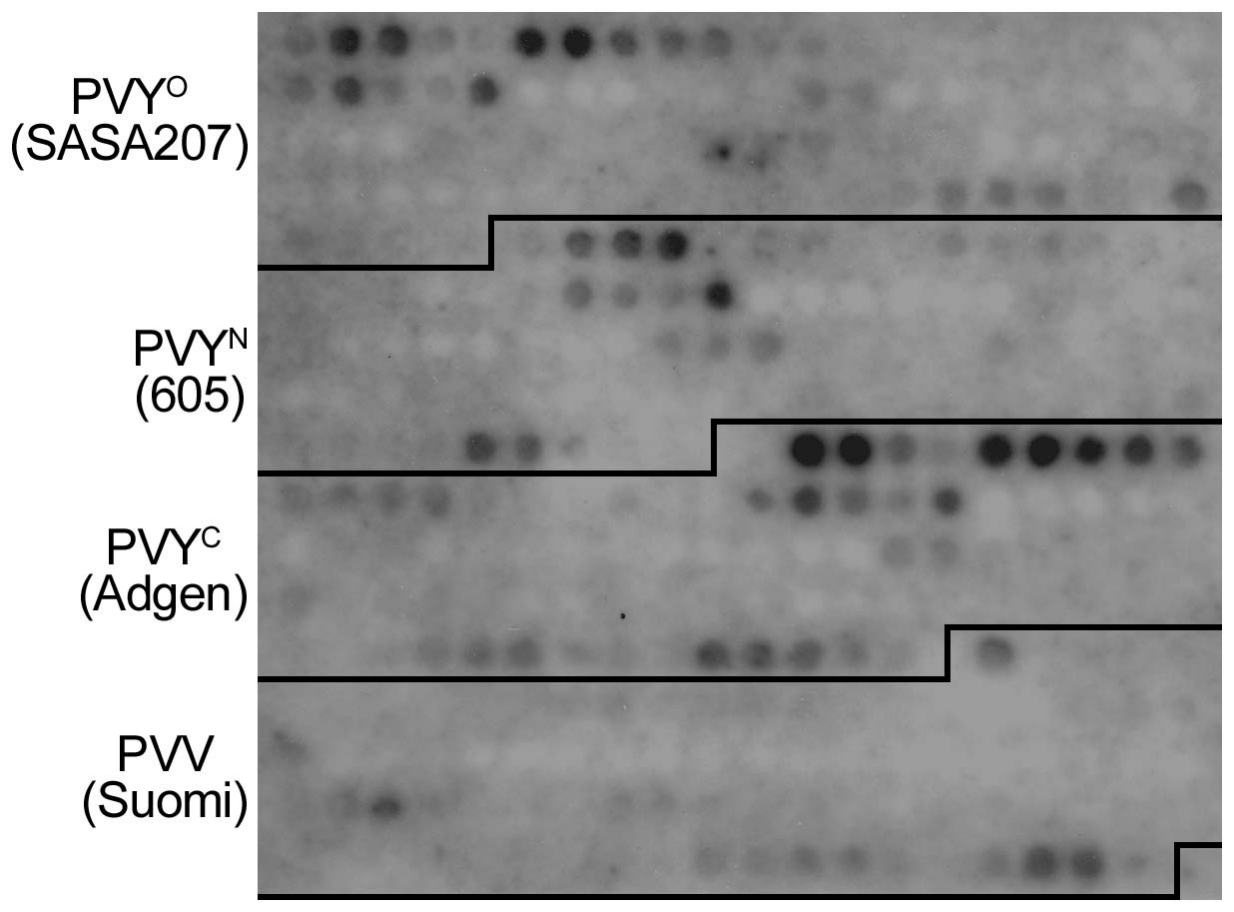
Detection of the PVY and PVV CP-derived peptides with polyclonal antibodies against CP of PVY. The membrane shown in Fig. 1 was probed with polyclonal rabbit anti-PVY CP antibodies and signals were detected using an alkaline phosphatase–conjugated pig anti-rabbit antibody. The membrane sections containing the peptides of different viruses are framed.

The membrane was also probed with polyclonal rabbit antibodies raised against PVY virions. The strongest reactivity was against peptides representing the N-terminal-most 50 aa of CP in all strains of PVY (Fig. 2). A few peptides corresponding to other regions of CP, especially the C-terminus, were also detected, but signals were weaker. The polyclonal antibodies showed some, relatively weak cross-reactivity with peptides designed based on CP of PVV (Fig. 2).

### Alanine Scanning and Deletion Analysis Define the Minimal Epitopes

**Figure 3:**
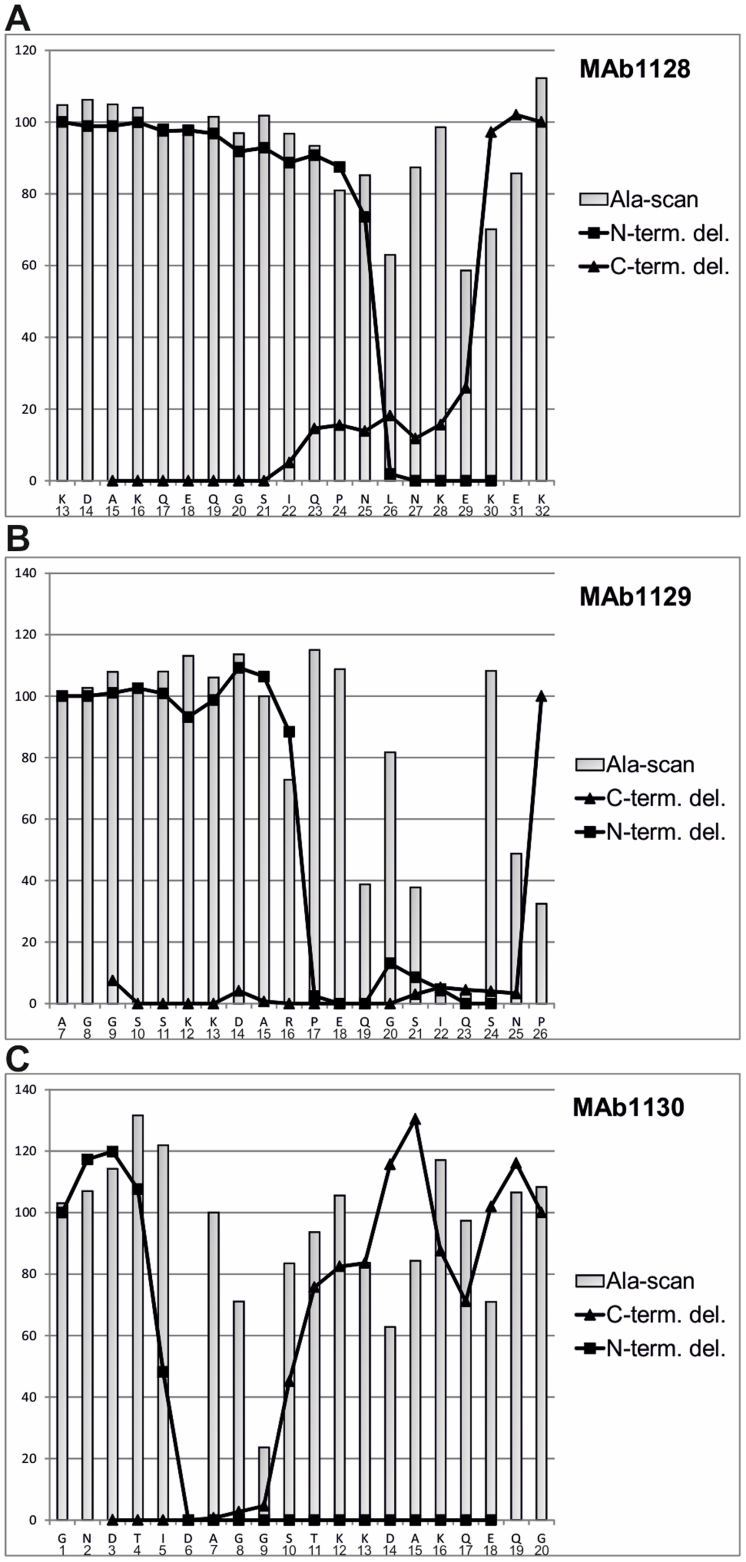
Fine-mapping of the MAb epitopes. Alanine scanning and deletion mapping of selected peptides were used to define the minimal epitope detected by each MAb. In alanine scanning, each residue at a time was replaced with an alanine, whereas in deletion mapping one residue at a time was removed from the N- or C-terminus of the peptide. The peptides subjected to the analyses were N-P5 (MAb1128), O-P3 (MAb1129), and N-P1 (MAb1130) shown in Fig. 1. Probing of the peptides with the MAbs was done as in Fig. 1. The chemiluminescence signals were quantified. Each bar and line represents the relative signal intensity compared with the corresponding wild-type peptide.

The epitope-related peptides identified in the SPOT analysis were further analyzed to yield a fine map for each epitope. During alanine scanning mutagenesis, one residue at a time was replaced with an alanine, whereas in the C- and N-terminal deletion analysis, aa residues were removed one by one (S1 Fig.). The peptides analyzed, as identified by peptide screening (Fig. 1), were N-P5 (^13^KDAKQEQGSIQPNLNKEKEK^32^) recognized by MAb1128, O-P3 (^7^AGGSSKKDARPEQGSIQSNP^26^) recognized by MAb1129, and NP-1 (^1^GNDTIDAGGSTKKDAKQEQG^20^) recognized by MAb1130 (Fig. 3). Results obtained in the analysis of the aforementioned peptides were verified by analyzing another peptide detected with the MAb, as illustrated in S1 Fig. The new SPOT peptide array containing the mutated and shortened peptides was probed with MAb1128, MAb1129, and MAb1130. The C- and N-terminal deletions demonstrated that the minimal epitope of MAb1128 was ^25^NLNKEK^30^ (Fig. 3). Alanine scanning further showed that replacement of ^25^N or ^27^N with alanine weakened the recognition by MAb1128, and replacement of ^26^L, ^29^E, or ^30^K nearly precluded recognition. For MAb1129, the minimal epitope was ^16^RPEQGSIQSNP^26^ (Fig. 3). The most critical aa residues for recognition by MAb1129 were ^22^I and ^23^Q. The epitope of MAb1130 was close to the N-terminus of CP and defined by residues ^5^IDAGGS^10^ (Fig. 3). Recognition of the peptide by MAb1130 was abrogated by mutation of residue ^6^D and strongly reduced by mutation of ^9^G (Fig. 3).

Epitope mapping in the aforementioned analysis was done using only three PVY strains. Therefore, the 60 most N-terminal CP residues of 369 PVY strains whose sequences are available in the curated Description of Plant Viruses database (http://www.dpvweb.net/) [40] were aligned to assess the conservation of the epitopes we identified. The variants are listed in Table 1, and the actual aa sequences are aligned in S1 Table. The MAb1128 epitope, ^25^NLNKEK^30^, was conserved in most PVY isolates designated to PVY^N^ strains and variants, such NTN strains, but it was not found in the isolates designated to PVY^O^ or PVY^C^ strain groups. The epitope of MAb1130 was also quite conserved in the large majority of PVY isolates. The greatest variability was observed in the epitope of MAb1129 (Table 1).

**Table 1:** Variable amino acids detected in the PVY CP epitope sequences of PVY isolates obtained from the curated Descriptions of Plant Viruses database (http://www.dpvweb.net/).

MAb1130 (O, C, N)^a^		Mab1129 (O, C)^a^		MAb1128 (N)^a^	
Epitope^b^	n^c^	Epitope^b^	n^c^	Epitope^b^	n^c^
I***D***AGGS	322	RPEQGS***IQ***SNP	90	NL***N***KE***K***	155
IDAG**E**S	11	**K**PEQGSIQ**P**NP	21	**S**LNKEK	20
IDAGG**N**	10	**K**PEQGSIQSNP	10	**I**LNKEK	4
IDAG**EN**	10	**K**PEQGSIQSN**L**	8	N**P**NKEK	3
ID**T**GGS	3	**K**PEQGSIQ**L**NP	7	NLNK**G**K	2
IDAGG**G**	2	**K**PEQGSIQ**R**NP	6	**K**LNKEK	2
ID**T**GG**N**	1	RPEQGSIQSN**L**	5	N**F**NKEK	2
IDA**V**G**D**	1	**KQ**EQGSIQ**P**N**T**	4	NL**I**KEK	1
IDA**VE**S	1	**K**PEQGSIQ**PK**P	3	NLNN**G**K	1
IDAG**EK**	1	**K**PEQSSIQSNP	3	N**P**NK**R**K	1
I**N**AGGS	1	**KL**EQGSIQ**P**NP	2	N**H**NKEK	1
IDAG**VN**	1	**K**PEQ**D**SIQ**PSS**	2	NLNKE**E**	1
IDAGG**I**	1	**K**PEQGSIQ**PS**P	2	NL**S**KEK	1
IDA**R**G**T**	1	**K**PEQGSIQ**PSS**	2		
**N**DAGGS	1	**K**PEQSSIQSN**L**	2		
IDAGG**D**	1	**KL**EQ**D**SIQSNP	1		
**MH**AGGS	1	**K**PAQGSIQ**PS**P	1		
		**K**PEQGSIQ**PT**P	1		
		**K**PEQGSIQSN**Q**	1		
		**K**PEQGSIQSN**S**	1		
		R**L**EQGSIQSNP	1		
		RPE**P**GSIQSNP	1		
		RPEQ**S**SIQSNP	1		
Total	369		175		194
					

a Distribution of the epitope in PVY strain groups. O, C and N refer to the PVY strain groups PVY^O^, PVY^C^, and PVY^N^, respectively.

b Substitution of the highlighted residues (bold, underlined letters in italic) for alanine in the epitope sequence shown in the uppermost line abolished detection of the protein by the MAb. The bold and underlined residues in other sequences represent natural variability of the epitope sequence in PVY isolates.

c n indicates the number of PVY CP-encoding sequences analyzed.

### Influence of Mutations in CP Epitopes on MAb Detection

Alanine scanning of peptides indicated that certain aa substitutions would lead to a lack of recognition of the correspondingly mutated CP by one or several MAbs. To verify these results using intact CP, His_6_-tagged wild-type and three mutated CPs of PVY^N^-605 and one mutated CP of PVY^O^-UK were expressed in *Escherichia coli.* The bacterial lysates were tested by western blot analysis using each of the MAbs.

**Figure 4:**
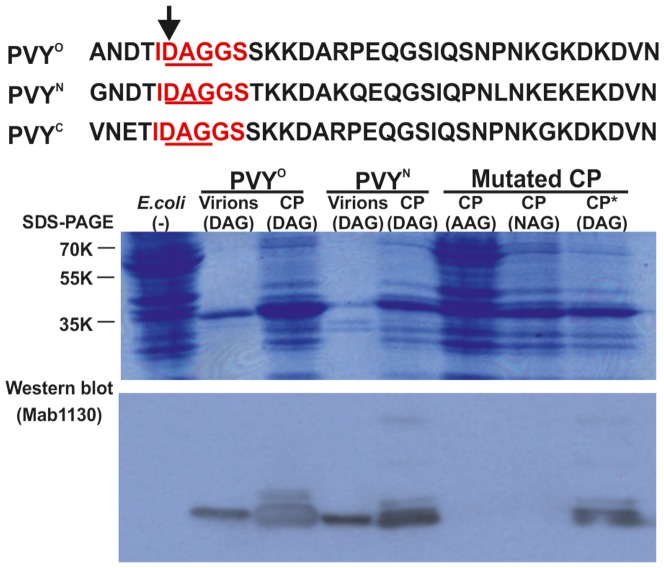
Effects of mutations in the conserved DAG motif of PVY CP on recognition with MAb1130. The N-terminal 35 residues of CP in PVY^O^-SASA207, PVY^N^-605 and PVY^C^-Adgen are aligned, and the residue D6, which was mutated, is indicated by an arrow. Red highlighting denotes the minimal epitope recognized by MAb1130, and the conserved DAG motif is underlined. Crude proteins extracted from *E. coli* expressing an individual recombinant PVY^N^-605 CP or mutant were analyzed by SDS-PAGE and stained with Coomassie Blue to detect the proteins. Positions of molecular size markers (in kilodaltons) are shown to the left. Virions of the wild-type viruses PVY^N^-605 and PVY^O^-UK and proteins of *E. coli* transformed with an empty expression vector were included as positive and negative controls, respectively. DAG, AAG, and NAG indicate the bacterial lysates containing the recombinant CPs of the wild-type PVY^N^-605 and CPs carrying substitution D6A or D6N, respectively (wild-type CP carrying the DAG motif is marked with an asterisk). The corresponding western blot using MAb1130 is shown below the gel.

**Figure 5:**
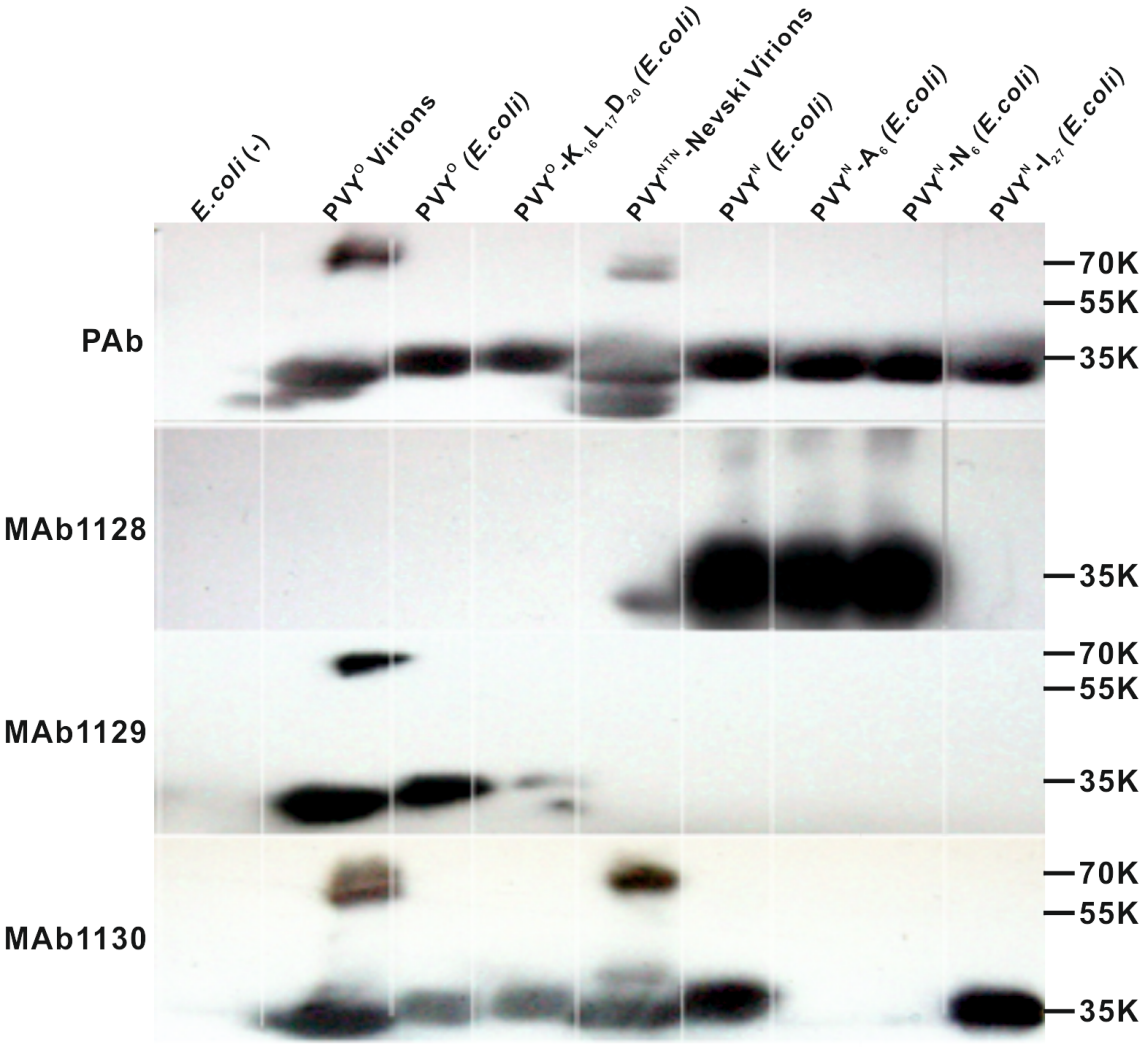
Recognition of PVY CP and mutants by the polyclonal antibody or various MAbs differs depending on specific mutations in the CP. Crude proteins extracted from *E. coli* expressing the recombinant PVY^O^-UK or PVY^N^-605 CP and their mutants were analyzed by western blotting using a polyclonal antiserum (PAb) and the three MAbs against PVY CP. Positions of molecular size markers (in kilodaltons) are shown to the right. Virions of the wild-type viruses PVY^O^-UK and PVY^N^-NTN Nevski, and proteins of *E. coli* transformed with an empty expression vector [*E. coli* (-)] were included as positive and negative controls, respectively. The amino acid substitutions introduced to CPs and their positions are specified in the names of the mutant proteins shown at the top of the blot. Detection of virions with PAb (and PVY^O^-UK virions with MAb1129 and MAb1130) revealed dimers (ca. 70 kDa) of the CP besides the expected monomers (ca. 35 kDa), indicating the CP-CP interactions essential for virion formation were not fully denatured by the SDS treatment.

Alanine scanning predicted that the substitution D6A would abolish recognition of PVY CP by MAb1130 (Fig. 3), and this result was verified by PVY^N^-605 showing the lack of the Mab1130::CP interaction (Fig. 4). The substitution D6N has been reported in a PVY isolate described from tobacco (NCBI accession no. X68222 [41]), and this mutation introduced to CP of PVY^N^-605 also abolished detection with MAb1130 (Fig. 4). However, both aforementioned CP mutants were detected with MAb1128 (Fig. 5).

Hidaka et al. [42] reported a PVY isolate with three aa substitutions (R16K, P17L, G20D); among these, R16A reduced detection of the peptide with MAb1129 (Fig. 3). Introduction of these three aa substitutions to CP of PVY^O^-UK greatly reduced the signals of detection in western blots (Fig. 5).

PVY^N^-NTN Nevski was isolated from potato cv. Nevski in Finland and causes tuber necrosis symptoms in inoculated potato cultivars (Y. Tian and J. Valkonen, unpublished data). This PVY strain contains an aa substitution N25T in CP (NCBI accession no. JX432967) in the first residue of the epitope of MAb1128. PVY^N^-NTN Nevski was detected with MAb1128 (Fig. 5, Table 1), however, signals were weaker than for PVY^N^-605.

Alanine scanning with peptide N-P5 suggested that substitution N27A may weaken the interaction between MAb1128 and PVY^N^ CP (Fig. 3). A PVY^N^ strain with the N27I mutation has been reported (U09508; [43]), and this substitution was introduced to CP of PVY^N^-605. The mutant CP was detected with MAb1130 but not with MAb1128 (Fig. 5).

The bacterial lysates used in these experiments were also tested with MAbs using triple-antibody sandwich (TAS)-ELISA, and results were consistent with western blot analyses (Table 2). All PVY CPs and mutants expressed in *E. coli* were detected with anti-PVY PAb by double-antibody sandwich (DAS)-ELISA (Table 2).

**Table 2:** Detection of recombinant PVY CP and CP mutants by DAS-ELISA and TAS-ELISA.

Proteins tested^a^	DAS-ELISA	TAS-ELISA		
	(PAb)	MAb1128	MAb1129	MAb1130
*E. coli* (total protein)	-	-	-	-
PVY^O^-UK (virions)	+	-	+	+
PVY^O^ (CP)	+	-	+	+
PVY^O^-K_16_L_17_D_20_ (CP)	+	-	-	+
PVY^NTN^ Nevski (virions)	+	+	-	+
PVY^N^ (CP)	+	+	-	+
PVY^N^-A_6_ (CP)	+	+	-	-
PVY^N^-N_6_ (CP)	+	+	-	-
PVY^N^-I_27_ (CP)	+	-	-	+

a Recombinant wild-type or mutated CPs of PVY^O^-UK and PVY^N^-605 were expressed in *E. coli*, and the bacterial cell lysate was used for detection of CP. Total protein from *E. coli* expressing no foreign protein and virions of PVY^O^-UK and PVY^NTN^ Nevski purified from tobacco plants were used as controls. Absorbance at 405 nm (*A*_405_) was recorded using a microtiter plate reader. The sample was regarded as positive (+) if the value was two times higher (± standard deviation) than that of the total protein from *E. coli* expressing no foreign protein used as a negative (−) control.

## Discussion

Detection of PVY is an integral part of the seed potato certification schemes [15]. Here, we characterized the epitopes of three commercially available MAbs used in the detection of the three common strain groups of PVY. The SPOT technique was utilized for epitope mapping and to pinpoint the critical residues required for PVY recognition [39]. The results were further verified using site-directed mutagenesis of full-length recombinant PVY CPs. All of the MAb epitopes we studied were mapped to the N-terminal 30 residues of CP. Similarly, previous studies localized the epitopes of six different MAbs to the first 31 [31] or 50 residues [15] of PVY CP. This region is the most exposed and variable part of the potyvirus CP [21]. As shown by multiple alignments beyond this region, the aa sequence becomes more conserved when it begins to form the helix, and owing to its inaccessibility becomes less immunogenic and less suitable for detection of specific PVY strains. Consequently, also the PAb bound preferentially to the N-terminal peptides of CP, confirming that the N-terminus is the most immunogenic portion of the PVY CP. Previous studies using three independent PAb produced by immunization of rabbits with virions of PVY^N^ found that PVY CP contains five immunodominant regions: two at the N-terminus (aa 1-25 and 29–39), two in the middle region (aa 70–95 and 163–170) and one at the C-terminus (aa 240–267) [44]. Also our studies revealed weak but detectable interaction of PAb with some of the C-terminal peptides. Furthermore, antibodies raised against peptides corresponding to the C-terminal region of PVY CP can detect the intact CP [45].

The minimal epitope for MAb1130, ^5^IDAGGS^10^, was common to all studied PVY strain groups. When sequences from 369 different PVY isolates were aligned, only a few differences in the epitope region were found, thus indicating that MAb1130 would probably recognize most isolates without being able to distinguish between them. Roughly 90% of the studied strains had no changes in the epitope sequence, and even more strains maintained the central portion of the epitope—the sequence DAG. This sequence was also present in the CP of the control PVV strain, and the corresponding peptide showed reactivity with the PAb against PVY. The conservation of this epitope among PVY strains reflects the important function of the DAG motif as it allows the transmission of PVY and related viruses from plant to plant by aphids [46]. Epitopes of other MAbs against PVY CP [15], [47] have been characterized and found to partially overlap with the ^5^IDAGGS^10^ sequence identified in our present study, revealing that this region is highly immunogenic in PVY, as reported also on other potyviruses (e.g., [37], [48], [49]).

The minimal epitope recognized by MAb1129 was ^16^RPEQGSIQSNP^26^ present in O- and C-strains of PVY. The peripheral portions of this sequence are highly variable, but the middle portion, namely ^18^EQGSIQ^23^, is conserved throughout the PVY^O^ and PVY^C^ strains we evaluated, with only very few exceptions (Table 1, S1 Table). In N-strains, this CP epitope region varies, and the peptides from this region did not bind MAb1129.

The minimal epitope recognized by MAb1128, ^25^NLNKEK^30^, was present only in the PVY^N^ strain CP. The corresponding sequence of PVY^O^ and PVY^C^ strains contained substitutions L26P and E29G, thus explaining why MAb1128 did not recognize these strains. According to the aa sequence alignment (S1 Table), most PVY isolates belonging to N-strain and the NTN variants contain this sequence and should therefore be detected by MAb1128. The epitopes of two other commercially available and commonly used PVY^N^-specific MAbs (SASA-N, and Neogen-N  =  MAb1051) have been localized to CP residues 22–30 [31] and hence overlap with the epitope of MAb1128. In the course of the study, we detected PVY^N^-Nevski with MAb1128 and MAb1051 (Neogen-N) in both DAS-ELISA and triple antibody sandwich (TAS) ELISA, whereas Neogen-N does not detect the strain PVY^NTN^-AST in TAS-ELISA according to a previous study [31]. Both PVY strains contain the minimal epitope ^25^NLNKEK^30^ of MAb1128. Therefore, the question whether or not MAb1128 and Neogen-N share the same epitope requires further study. Similar to Mab1128, substitution N27I prevents detection of the PVY^N^ CP with SASA-N and Neogen-N [31].

Designation of PVY strains to the strain groups N, O and C is commonly done based on detection with MAbs or by phylogenetic analysis of CP (nucleotide or deduced aa) sequences. However, strain group identification is actually based on the biological differences of the PVY strain groups in terms of their ability to overcome resistance genes in potato or cause severe symptoms in potato and tobacco. However, recent studies show that the determinants of the biological differences, which are used to recognize the strain groups of PVY isolates [12], reside in the helper component proteinase (HCpro) of PVY [50]–[52]. It is therefore remarkable how well the serogroups determined by anti-CP MAbs correlate with the biological differences of the strain groups determined by HCpro. Interactions between CP and HCpro are essential in vector transmission of potyviruses [53] and involve the DAG motif besides other regions of the CP [46], [54]. Furthermore, HCpro-CP interactions are probably essential during the infection cycle in host plants [55], [56]. Therefore, it is plausible that high selection pressure directs concerted evolution of HCpro and CP [55], which is reflected in the functional phenotypes of PVY isolates.

MAbs to PVY HCpro have been produced, including MAb 8E1 that reacts with the HCpro of O and C strains, and MAb 8B9 reacting with the HCpro of isolates from all three strain groups PVY including N strains [57]. It is possible that detection of PVY isolates with MAbs recognizing specific functional motifs of the multifunctional HCpro [50]–[52] could provide a higher resolution and more fine-tuned grouping of PVY isolates according to their biological differences. Furthermore, as proposed by Canto et al. [57] two decades ago, detection of PVY using MAbs to both HCpro and CP would be helpful for recognition of the highly virulent PVY^N^ Wilga (PVY^N:O^) strains that have recombined the whole HCpro or segments thereof from PVY^O^ [19], [58], [59]. They cause tuber necrosis and other severe symptoms in potato and are widely distributed [13].

Taken together, the detailed MAb epitope analysis provided in this study should allow a sequence-based identification of the PVY serotypes and to predict whether particular isolates could escape detection or whether they will be detected by specific anti-CP MAbs. Our results help explain why different PVY strain groups can be distinguished using these MAbs. Furthermore, the data may be used for the generation of novel tools for detection of different PVY strains.

## Materials and Methods

### Antibodies

PVY detection reagent set, which contains rabbit PAb (1130-2, Neogen Phytodiagnostics) for coating, MAbs (TAS-ELISA) or alkaline phosphatase conjugated MAbs (DAS-ELISA) for probing, and horseradish peroxidase–conjugated polyclonal rabbit anti-mouse immunoglobulins (TAS-ELISA) was used (Neogen). Also polyclonal porcine anti-rabbit immunoglobulins were used (Dako, Carpinteria, CA, USA). Mab1128, Mab1129, Mab1130 and the rabbit polyclonal antiserum against PVY were produced using whole viruses as immunogens (Neogen).

### Peptide Synthesis

Spot peptides [39] were synthesized with a fully automated MultiPep Spot robot (Intavis AG, Köln, Germany) on amino-polyethylene glycol–functionalized cellulose membranes (SynthoPlan APEG CE Membrane, AIMS Scientific Products, Product number AC-S01-12) via Fmoc chemistry. The PVY full-length CPs of different PVY strains were synthesized as 20-aa peptides (3-aa overlap), resulting in 84 peptides per PVY CP. The PVY strains were spotted on each membrane in the following order: PVY^O^ (isolate SASA207; GenBank accession number AJ584851; [60]), PVY^N^ (isolate 605; X97895; [61]), PVY^C^ (isolate Adgen; AJ890348; [62]), and PVV (isolate Suomi; AJ278894; [63]). The initially identified MAb epitopes were further characterized by subjecting the relevant peptides to alanine scanning (residues replaced one by one with alanine) and to N- or C-terminal deletion (residues removed one by one).

### Probing of SPOT Membranes

The assay was done as described [64]. Briefly, antibodies and conjugates were diluted with Tris-buffered saline containing 0.05% (v/v) Tween 20 and supplemented with 3.0% skim milk (blocking buffer). The MAbs were used at 1 µg/ml and polyclonal antisera at 1∶1000 dilutions in blocking buffer. Secondary antibody incubations were done at 1∶1000 dilutions in blocking buffer. Antibody binding was visualized using enhanced chemiluminescence and X-ray film.

### Mutagenesis and Expression of PVY CP in *E. coli*

Full-length PVY CP-encoding sequences were PCR-amplified with primers corresponding to the 5′-end and complementary to the 3′-end of PVY^N^605 and PVY^O^UK CP-encoding sequences and introducing *Bam*HI and *Pst*I restriction sites, respectively (Table 3). The amplified products were cloned into the expression vector pQE30 (Qiagen Hilden, Germany).

**Table 3:** Primer pairs used for mutagenesis of CP-encoding sequence of PVY^N^ or PVY^O^.

Primer	Sequence^a^	Tm °C	Purpose
PVY^N^-F	5′-CG*GGATCC*ggaaatgacacaatcgatgcag-3′	66.7	Amplification of PVY^N^ CP-encoding sequence
PVY^N^-R	5′-AA*CTGCAG*TCA catgttcttcactccaag-3′	60.8	
			
PVY^N^A_6_-F	5′-CG*GGATCC*ggaaatgacacaatcg**CA**gcaggaggaag-3′	77.0	Amino acid substitution D_6_A
PVY^N^N_6_-F	5′-CG*GGATCC*ggaaatgacacaatc**A**atgcaggaggaag-3′	73.8	Amino acid substitution D_6_N
			
PVY^N^I_27_-F	5′-P-caaccaaatctca**T**caaggaaaag-3′	64.8	Amino acid substitution N_27_I
PVY^N^I_27_-R	5′-P-aatgctaccttgctcttgttttg-3′	63.5	
			
PVY^O^-F	5′-CG*GGATCC*gcaaatgacacaattgatgcag-3′	65.6	Amplification of PVY^O^ CP-encoding sequence
PVY^O^-R	5′-AA*CTGCAG*TCA catgttctttactccaag-3′	57.4	
			
PVY^O^K_16_L_17_D_20_-F	5′-P-aaagatgcaa**A**ac**T**agagcaag**A**cagcatccag-3′	76.3	Amino acid substitutions R_16_K, P_17_L and Q_20_D
PVY^O^K_16_L_17_D_20_-R	5′-P-cttgctgcttcctcctgcatc-3′	71.6	

a Restriction sites are in italic and underlined. The nucleotides introduced before the restriction sites increase the efficiency of digestion. Mutated nucleotides are in bold type. “P” indicates that the 5′-terminus of the primer is phosphorylated. Tm indicates melting temperature in Celsius degrees.

Four mutated CPs were also constructed (Table 3) using the Phusion Site-Directed Mutagenesis kit (Finnzymes, Espoo, Finland). The aspartic acid at position 6 (I**D**AGGS epitope) of PVY^N^-605 CP was mutated to alanine and asparagine to correspond to the sequences of PVY^N^-^6^A and PVY^N^-^6^N, respectively. Asparagine at position 27 (NL**N**KIKE epitope) of PVY^N^-605 CP was mutated to isoleucine. Arginine, proline, and glycine at positions 16, 17, and 20 of PVY^O^-UK CP were mutated to lysine, leucine, and aspartic acid, respectively (PVY^O^ ^16^K^17^L^20^D). The CP of the PVY-Nevski isolate (^25^T instead of ^25^N) was also included in the study. All constructs generated were transformed into *E. coli* strain M15 (pREP4+) (Qiagen) for protein production.

Protein expression and purification was done as earlier [65]. Briefly, cells were harvested by centrifugation at 11,000×*g* for 10 min, freezed in liquid nitrogen, and lysed by grinding on ice. The resultant lysate was suspended in ELISA extraction buffer (phosphate-buffered saline including 0.05% (v/v) Tween 20 and 2% (w/v) polyvinylpyrrolidone, pH 7.4) and fractioned via centrifugation at 15,000×*g* for 30 min at 4°C. The supernatant was analyzed with DAS- and TAS-ELISA and with SDS-PAGE. The His-tagged proteins used in western blotting were purified using the Ni-NTA strategy (Qiagen, Hilden, Germany) and were analyzed by SDS-PAGE and western blotting.

## Supporting Information

S1 Fig **Alanine scanning and deletion mapping to define the minimal epitope detected by MAb1128 in PVY^O^–UK CP.**
(PDF)

S1 Table **Alignment of the 60 N-terminal residues of 369 PVY coat protein (CP) sequences obtained from the curated DPVweb database and analysis of the epitope sequences of MAb1128, MAb1129, and MAb1129.**
(PDF)
