# Supplementary material for: Analysis of Potato virus Y Coat Protein Epitopes Recognized by Three Commercial Monoclonal Antibodies
Source: PLoS One. 2014 Dec 26;9(12):e115766. doi: 10.1371/journal.pone.0115766 (PMC4277358; doi:10.1371/journal.pone.0115766)
Supplement: S1 Fig — Alanine scanning and deletion mapping to define the minimal epitope detected by MAb1128 in PVYO–UK CP. (PDF) [file pone.0115766.s001.pdf]

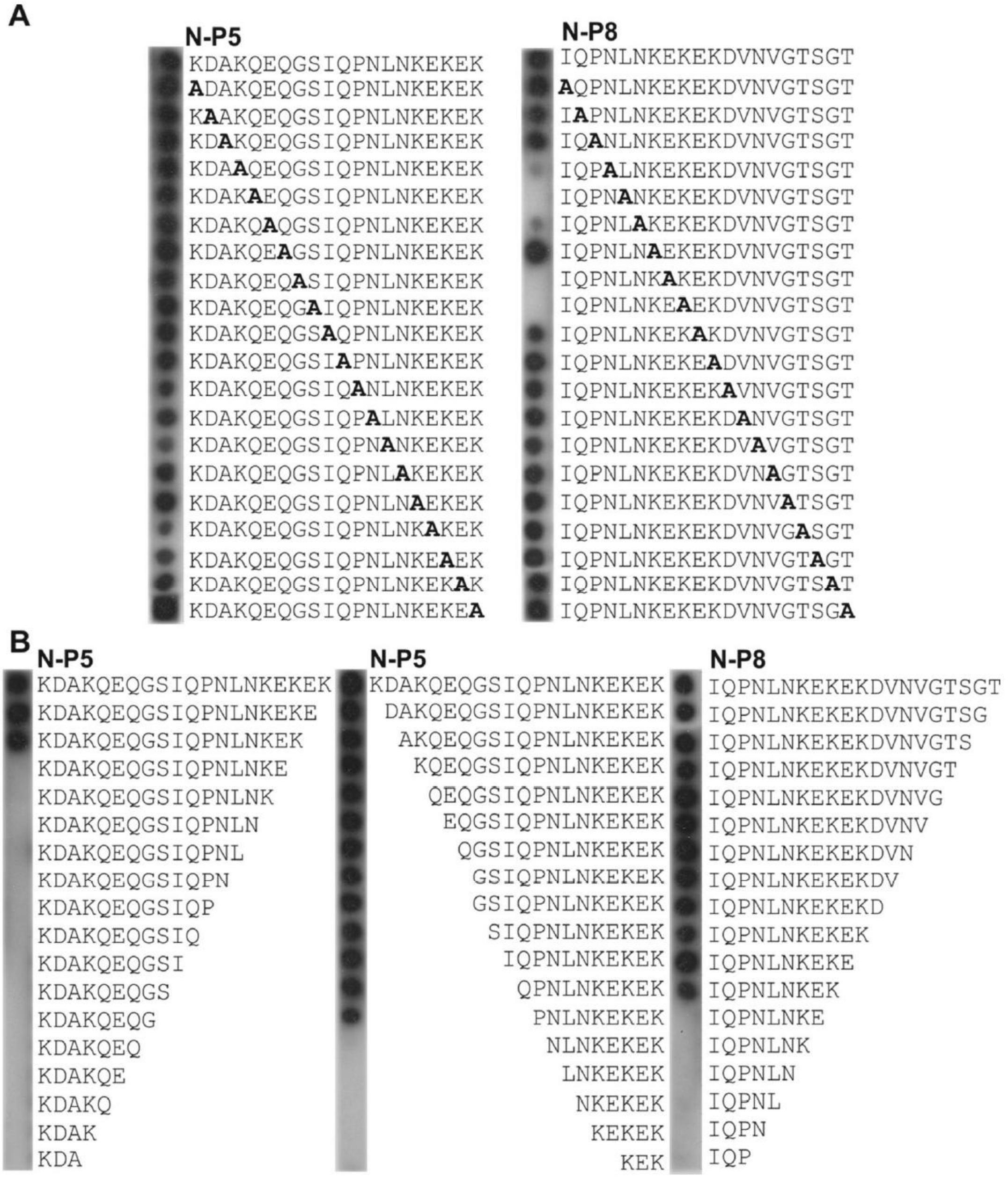

Supporting Figure S1. **A**, Alanine scanning mutagenesis and **B**, C- and N-terminal deletion analyses of peptides N-P5 and N-P8 to determine the minimal epitope recognized by MAb1128. In alanine scanning mutagenesis, one residue at a time was replaced with an alanine, whereas in the C- and N-terminal deletion analysis, aa residues were removed one by one.
