# Supplementary material for: Analysis of Potato virus Y Coat Protein Epitopes Recognized by Three Commercial Monoclonal Antibodies
Source: PLoS One. 2014 Dec 26;9(12):e115766. doi: 10.1371/journal.pone.0115766 (PMC4277358; doi:10.1371/journal.pone.0115766)
Supplement: S1 Table — Alignment of the 60 N-terminal residues of 369 PVY coat protein (CP) sequences obtained from the curated DPVweb database and analysis of the epitope sequences of MAb1128, MAb1129, and MAb1129. (PDF) [file pone.0115766.s002.pdf]

Supplementary Table S1. Alignment of the 60 N-terminal amino acid residues of 369 PVY coat protein (CP) sequences obtained from the curated DPVweb database and analysis of the epitope sequences of MAbs 1128, 1129, and 1129.

Table S1 (A) Includes the PVY isolates containing the minimal CP epitope recognized by MAb1128 (typically PVY^N isolates)
Table S1 (B) Includes the PVY isolates containing the minimal CP epitope recognized by MAb1129 (PVY^O and PVY^C isolates).
Table S1 (C). Isolates containing significant variation within the epitope recognized by MAb1128.
Table S1 (D). Isolates containing significant variation within the epitope recognized by MAb1129.

<u>The strain group</u> announced for each PVY isolate is **(a)** tentative and based on the claim of the author made in the sequence database, **(b)** determined based on the proper biological experiments, or **(c)** tentative based on phylogenetic grouping of the isolate in the analysis of PVY CP sequences. These three different criteria are indicated by **superscripts a, b and c**, respectively.

<u>The columns to the left provide predictions</u> regarding detection of each CP by the MAb1128, MAb1129, and MAb1130. Plus (+) indicates that the isolate contains the minimal epitope sequence of the respective MAb, whereas asterisk (*) indicates that the corresponding epitope sequence contains substitutions or is missing.

Table S1 (A). The epitope recognized by MAb1128 is shown in blue. The epitope recognized by MAb1130 is shown in red. The protein domain shown in pink is highly conserved and differs from the epitope recognized by MAb1129. Amino acid substitutions within these regions are shown in black (underlined).

Accession number	N'-terminal residues 1-60	Strain group	MAb 1128	MAb 1129	MAb 1130
ab025415	GNDT**IDAGGS**TKKDA**KQEQGSIQPNLNKEK**EKDVNVGTSGTHTVPRIKAITSKMRMPKSK	N[a]	+	*	+
ab025416	GNDT**IDAGGS**TKKDA**KQEQGSIQPNLNKEK**EKDVNVGTSGTHTVPRIKAITSKMRMPKSK	N[a]	+	*	+
ab025417	GNDT**IDAGGS**TKKDA**KQEQGSIQPNLNKEK**EKDVNVGTSGTHTVPRIKAITSKMRMPKSK	N[a]	+	*	+
ab042811	GNDT**IDAGGS**TKKDA**KQEQGSIQPNLNKEK**EKDVNVGTSGTHTVPRIKAITSKMRMPKSK	N[a]	+	*	+
ab042812	GNDT**IDAGGS**TKKDA**KQEQGSIQPNLNKEK**EKDVNVGTSGTHTVPRIKAITSKMRMPKSK	N[a]	+	*	+
ab042813	GNDT**IDAGGS**TKKDA**KQEQGSIQPNLNKEK**EKDVNVGTSGTHTVPRIKAITSKMRMPKSK	N[a]	+	*	+
ab295477	GNDT**IDAGGS**TKKDA**KQEQGSIQPNLNKEK**EKDVNVGTSGTHTVPRIKAITSKMRMPKSK	N[a]	+	*	+
ab295478	GNDT**IDAGGS**TKKDA**KQEQGSIQPNLNKEK**EKDVNVGTSGTHTVPRIKAITSKMRMPKSK	NTN[a]	+	*	+
ab295479	GNDT**IDAGGS**TKKDA**KQEQGSIQPNLNKEK**EKDVNVGTSGTHTVPRIKAITSKMRMPKSK	NTN[a]	+	*	+
ab331515	GNDT**IDAGGS**TKKDA**KQEQGSIQPNLNKEK**EKDVNVGTSGTHTVPRIKAITSKMRMPKSK	NTN[c]	+	*	+
ab331516	GNDT**IDAGGS**TKKDA**KQEQGSIQPNLNKEK**EKDVNVGTSGTHTVPRIKAITSKMRMPKSK	NTN[c]	+	*	+
ab331517	GNDT**IDAGGS**TKKDA**KQEQGSIQPNLNKEK**EKDVNVGTSGTHTVPRIKAITSKMRMPKSK	NTN[c]	+	*	+

ab331518	GNDTIDAGGSTKKDAKQEQGSIQPNLNKEKEKDVNVGTSGTHTVPRIKAITSKMRMPKSK	NTN[c]	+	*	+
ab331519	GNDTIDAGGSTKKDAKQEQGSIQPNLNKEKEKDVNVGTSGTHTVPRIKAITSKMRMPKSK	NTN[c]	+	*	+
ab331538	GNDTIDAGGSTKKDAKQEQGSIQPNLNKEKEKDVNVGTSGTHTVPRIKAITSKMRMPKSK	NTN[c]	+	*	+
ab331539	GNDTIDAGGSTKKDAKQEQGSIQPNLNKEKEKDVNVGTSGTHTVPRIKAITSKMRMPKSK	NTN[c]	+	*	+
ab331540	GNDTIDAGGSTKKDAKREQGSIQPNLNKEKEKDVNVGTSGTHTVPRIKAITSKMRMPKSK	NTN[c]	+	*	+
ab331541	GNDTIDAGGSTKKDAKQEQGSIQPNLNKEKEKDVNVGTSGTHTVPRIKAITSKMRMPKSK	NTN[c]	+	*	+
ab331542	GNDTIDAGGSTKKDAKQEQGSIQPNLNKEKEKDVNVGTSGTHTVPRIKAITSKMRMPKSK	NTN[c]	+	*	+
ab331543	GNDTIDAGGSTKKDAKQEQGSIQPNLNKEKEKDVNVGTSGTHTVPRIKAITSKMRMPKSK	NTN[c]	+	*	+
ab331544	GNDTIDAGGSTKKDAKQEQGSIQPNLNKEKEKDVNVGTSGTHTVPRIKAITSKMKMPKSK	NTN[c]	+	*	+
ab331545	GNDTIDAGGSTKKDAKQEQGSIQPNLNKEKEKDVNVGTSGTHTVPRIKAITSKMRMPKSK	NTN[c]	+	*	+
ab331546	GNDTIDAGGSTKKDAKQEQGSIQPNLNKEKEKDVNVGTSGTHTVPRIKAITSKMRMPKSK	NTN[c]	+	*	+
ab331547	GNDTIDAGGSTKKDAKQEQGSIQPNLNKEKEKDVNVGTSGTHTVPRIKAITSKMRMPKSK	NTN[c]	+	*	+
ab331548	GNDTIDAGGSTKKDAKQEQGSIQPNLNKEKEKDVNVGTSGTHTVPRIKAITSKMRMPKSK	NTN[c]	+	*	+
ab331549	GNDTIDAGGSTKKDAKQEQGSIQPNLNKEKEKDVNVGTSGTHTVPRIKAITSKMRMPKSK	NTN[c]	+	*	+
ab331550	GNDTIDAGGSTKKDAKQEQGSIQPNLNKEKEKDVNVGTSGTHTVPRIKAITSKMRMPKSK	NTN[c]	+	*	+
af126258	GNDTIDAGGSTKKDAKQEQGSIQPNLNKEKEKDVNVGTSGTHTVPRIKAITSKMKMPKSK	N[a]	+	*	+
af325928	GNDTIDAGGSTKKDAKQEQGSIQPNLNKEKEKDVNVGTSGTHTVPRIKAITSKMRMPKSK	N[b]	+	*	+
aj133454	GNDTIDAGGSTKKDAKQEQGSIQPNLNKEKEKDVNVGTSGTHTVPRIKAITSKMRMPKSK	N[b]	+	*	+
aj223592	GNDTIDAGGSTKKDAKQEQGSIQPNLNKEKEKDVNVGTSGTHTVPRIKAITSKMRMPKSK	N[b]	+	*	+
aj390285	GNDTIDAGGSTKKDAKQEQGSIQPNLNKEKEKDVNVGTSGTHTVPRIKAITSKMRMPKSK	N[b]	+	*	+
aj390286	GNDTIDAGGSTKKDAKQEQGSIQPNLNKEKEKDVNVGTSGTHTVPRIKAITSKMRMPKSK	N[b]	+	*	+
aj390287	GNDTIDAGGSSKKDAKQEQGSIQPNLNKEKEKDVNVGTSGTHTVPRIKAITSKMRMPKSK	N[b]	+	*	+
aj390288	GNDTIDAGGSTKKDAKQEQGSIQPNLNKEKEKDVNVGTSGTHTVPRIKAITSKMRMPKSK	N[b]	+	*	+
aj390289	GNDTIDAGGSTKKDAKQEQGIIQPNLNKEKEKDVNVGTSGTHTVPRIKAITSKMRMPKSK	N[b]	+	*	+
aj390291	GNDTIDAGGSTKKDAKQEQGSIQPNLNKEKEKDVNVGTSGTHTVPRIKAITSKMRMPKSK	N[b]	+	*	+
aj390293	GNDTIDAGGSTKKDAKQEQGSIQLNLNKEKEKDVNVGTSGTHTVPRIKAITSKMRMPKSK	N[b]	+	*	+
aj390294	GNDTIDAGGSTKKDAKQEQGSIQPNLNKEKEKDVNVGTSGTHTVPRIKAITSKMRMPKSK	N[b]	+	*	+
aj390298	GNDTIDAGGSTKKDAKQEQGSIQPNLNKEKEKDVNVGTSGTHTVPRIKAITSKMRMPKSK	N[b]	+	*	+
aj390299	GNDTIDAGGSTKKDAKQEQGSIQPNLNKEKEKDVNVGTSGTHTVPRIKAITSKMRMPKSK	N[b]	+	*	+
aj390300	GNDTIDAGGSTKKDAKQEQGSIQLNLNKEKEKDVNVGTSGTHTVPRIKAITSKMRMPKSK	N[b]	+	*	+
aj390303	GNDTIDAGGSTKKDAKQEQGSIQPNLNKEKEKDVNVGTSGTHTVPRIKAITSKMRMPKSK	N[b]	+	*	+
aj390308	GNDTIDAGGSTKKDAKQGQGSIQPNLNKEKEKDVNVGTSGTHTVPRIKAITSKMRMPKSK	N[b]	+	*	+

aj390309	GNDTIDAGGSTKKDAKQEQGSIQPNLNKEKEKDVNVGTSGTHTVPRIKAITSKMRMPKSK	N[b]	+	*	+
aj535662	GNDTIDAGGSTKKDAKQEQGSIQPNLNKEKEKDVNVGTSGTHTVPRIKAIASKMRMPKSK	NTN[a]	+	*	+
aj585197	GNDTIDAGGSTKKDAKQEQGSIQPNLNKEKEKDVNVGTSGTHTVPRIKAITSKMRMPKSK	N[b]	+	*	+
aj585198	GNDTIDAGGSTKKDAKQEQGSIQPNLNKEKEKDVNVGTSGTHTVPRIKAITSKMRMPKSK	N[b]	+	*	+
aj585342	GNDTIDAGGSTKKDAKQEQGSIQPNLNKEKEKDVNVGTSGTHTVPRIKAITSKMRMPKSK	N[b]	+	*	+
aj889866	GNDTIDAGGSTKKDAKQELGSIQPNLNKEKEKDVNVGTSGTHTVPRIKAITSKMRMPKSK	NTN[c]	+	*	+
aj890342	GNDTIDAGGSTKKDAKQELGSIQPNLNKEKEKDVNVGTSGTHTVPRIKAITSKMRMPKSK	NTN[c]	+	*	+
aj890343	GNDTIDAGGSTKKDAKQELGSIQPNLNKEKEKDVNVGTSGTHTVPRIKAITSKMRMPKSK	NTN[c]	+	*	+
aj890344	GNDTIDAGGSTKKDAKQEQGIIQPNLNKEKEKDVNVGTSGTHTVPRIKAITSKMRMPKSK	NTN[c]	+	*	+
aj890345	GNDTIDAGGSTKKDAKQEQGSIQPNLNKEKEKDVNVGTSGTHTVPRIKAITSKMRMPKSK	NTN[c]	+	*	+
aj890346	GNDTIDAGGSTKKDAKQEQGSIQLNLNKEKEKDVNVGTSGTHTVPRIKAITSKMRMPKSK	NTN[c]	+	*	+
aj890347	GNDTIDAGGSTKKDAKQEQGSIQPNLNKEKEKDVNVGTSGTHTVPRIKAITSKMRMPKSK	NTN[c]	+	*	+
am236792	ANDTIDAGGSNKKDAKQEQGSIQPNLNKEKEKDVNVGTSGTHTVPRIKAITSKMRMPKSK	N[b]	+	*	+
am236796	VNDTIDAGGSNKKDAKQEQGSIQPNLNKEKEKDVNVGASGTHTVPRIKAITSKMRMPKSK	N[b]	+	*	+
am236797	GNDTIDAGGSTKKDAKQEQGSIQPNLNKEKEKDVNVGTSGTHTVPRIKAITSKMRMPKSK	N[b]	+	*	+
am236799	VKDTIDAGGSNKKDAKQEQGSIQPNLNKEKEKDVNVGTSGTHTVPRIKAITSKMRMPKSK	N[b]	+	*	+
am236810	ANDTIDAGGSTKKDAKQEQGSIQQNLNKEKEKDVNVGTSGTHTVPRIKAITSKMKMPKSK	N[b]	+	*	+
am236814	GNETIDAGGSTKKDAKQEQGSIQPNLNKEKEKDVNVGTSGTHTVPRIKAITSKMRMPKSK	N[b]	+	*	+
am411502	GNDTIDAGGSTKKDAKQEQGSIQPNLNKEKEKDVNVGTSGTYTVPRIKAITSKMRMPKSK	*	+	*	+
am411503	GNDTIDAGGSTKKEAKQEQGSIQPNLNKEKEKDVNVGTSGTHTVPRIKAITSKMRMPKSK	*	+	*	+
am931254	GNDTIDAGGSTKKDAKQEQGSIQPNLNKEKEKDVNVGTSGTHTVPRIKAITSKMRMPRSK	*	+	*	+
ay166867	GNDTIDAGGSAKKDAKQEQGSIQPNLNKEKEKDVNVGTSGTHTVPRIKAITSKMRMPKSK	N[b]	+	*	+
ay841258	ANDTIDAGGSNKKDAKQEQGSIQPNLNKEKEKDVNVGTSGTHTVPRIKAITSKMRMPKSK	N[b]	+	*	+
ay841260	ANDTIDAGGSSKKDAKQEQGSIQQNLNKEKEKDVNVGTSGTHTVPRIKAITSKMRMPKSK	N[b]	+	*	+
ay841267	ANDTIDAGGSNKKDAKQEQGSIQPNLNKEKEKDVNVGTSGTHTVPRIKAITSKMRMPKSK	N[b]	+	*	+
ay841268	ANDTIDAGGSTKKDAKQEQGSIQQNLNKEKEKDVNVGTSGTHTVPRIKAITSKMRMPKSK	N[b]	+	*	+
ay884983	GNDTIDAGGSTKKDAKQEQGSIQPNLNKEKEKDVNVGTSGTHTVPRIKAITSKMRMPKSK	N[b]	+	*	+
ay884984	GNDTIDAGGSTKKDAKQEQGIIQPNLNKEKEKDVNVGTSGTHTVPRIKAITSKMRMPKSK	NTN[b]	+	*	+
d12570	GNDTIDAGGSTKKDAKQEQGSIQPNLNKEKEKDVNVGTSGTHTVTRIKAITSKMRMPKSK	N[a]	+	*	+
dq157180	GNDTIDAGGSNKKDAKQEQGSIQPNLNKEKEKDVNVGTSGTHTVPRIKAITSKMRMPKSK	N[c]	+	*	+
ef016294	GNDTIDAGGSTKKDAKQEQGIIQPNLNKEKEKDVNVGTSGTHTVPRIKAITSKMRMPKSK	NTN[a]	+	*	+
ef026075	GNDTIDAGGSTKKDAKQEQGSIQPNLNKEKEKDVNVGTSGTHTVPRIKAITSKMRMPKSK	N[b]	+	*	+

ef027859	GNDTIDAGGSTKKDAKQEQGSIQPNLNKEKEKDVNVGTSGTHTVPRIKAITSKMRMPKSK	N[a]	+	*	+
ef027860	GNDTIDAGGSTKKDAKQEQGSIQPNLNKEKEKDVNVGTSGTHTVPRIKAITSKMRMPKSK	NTN[b]	+	*	+
ef027861	GNDTIDAGGSTKKDVKQEQGSIQPNLNKEKEKDVNVGTSGTHTVPRIKAITSKMRMPKSK	N[a]	+	*	+
ef027862	GNDTIDAGGSTKKDAKQEQGSIQPNLNKEKEKDVNVGTSGTHTVPRIKAITSKMRMPKSK	NTN[a]	+	*	+
ef027863	GNDTIDAGGSTKKDAKQEQGSIQPNLNKEKEKDVNVGTSGTHTVPRIKAITSKMRMPKSK	NTN[a]	+	*	+
ef027864	GNDTIDAGGSTKKDAKQEQGSIQPNLNKEKEKDVNVGTSGTHTVPRIKAITSKMRMPKSK	NTN[a]	+	*	+
ef027865	GNDTIDAGGSTKKDAKQEQGSIQPNLNKEKEKDVNVGTSGTHTVPRIKAITSKMRMPKSK	NTN[a]	+	*	+
ef027868	GNDTIDAGGSTKKDAKQEQGSIQPNLNKEKEKDVNVGTSGTHTVPRIKAITSKMRMPKSK	N[a]	+	*	+
ef027869	GNDTIDAGGSTKKDAKQEQGSIQPNLNKEKEKDVNVGTSGTHTVPRIKAITSKMRMPKSK	NTN[a]	+	*	+
ef027879	GNDTIDAGGSTKKDAKQEQGSIQPNLNKEKEKDVNVGTSGTHTVPRIKAITSKMRMPKSK	NTN[a]	+	*	+
ef027880	GNDTIDAGGSTKKDAKQEQGSIQPNLNKEKEKDVNVGTSGTHTVPRIKAITSKMRMPKSK	NTN[a]	+	*	+
ef027881	GNDTIDAGGSTKKDAKQEQGSIQPNLNKEKEKDVNVGTSGTHTVPRIKAITSKMRMPKSK	NTN[a]	+	*	+
ef027882	GNDTIDAGGSTKKDAKQEQGIIQPNLNKEKEKDVNVGTSGTHTVPRIKAITSKMRMPKSK	NTN[a]	+	*	+
ef027886	GNDTIDAGGSTKKDAKQEQGSIQTNLNKEKEKDVNVGTSGTHTVPRIKAITSKMRMPKSK	N[a]	+	*	+
ef027891	GNDTIDAGGSTKKDAKQEQGSIQPNLNKEKEKDVNVGTSGTHTVPRIKAITSKMRMPKSK	N[a]	+	*	+
ef027892	GNDTIDAGGSTKKDAKQEQGSIQPNLNKEKEKDVNVGTSGTHTVPRIKAITSKMRMPKSK	NTN[a]	+	*	+
ef027897	GNDTIDAGGSTKKDAKQEQGSIQPNLNKEKEKDVNVGTSGTHTVPRIKAITSKMRMPKSK	NTN[a]	+	*	+
ef027898	GNDTIDAGGSTKKDAKQEQGSIQPNLNKEKEKDVNVGTSGTHTVPRIKAITSKMRMPKSK	NTN[a]	+	*	+
ef027899	GNDTIDAGGSTKKDAKQEQGSXQPNLNKEKEKDVNVGTSGTHTVPRIKAITSKMRMPKSK	NTN[a]	+	*	+
ef027901	GNDTIDAGGSTKKDAKQEQGSIQPNLNKEKEKDVNVGTSGTHTVPRIKAITSKMRMPKSK	NTN[a]	+	*	+
eu073855	ANDTIDAGGSTKKDAKQEQGSIQPNLNKEKEKDVNVGTSGTHTVPRIKAITSKMRMPKSK	*	+	*	+
eu073856	ANDTIDAGGSTKKDAKQEQGSIQPNLNKEKEKDVNVGTSGTHTVPRIKAITSKMRMPKSK	*	+	*	+
eu073857	ANDTIDAGGSTKKDAKQEQGSIQPNLNKEKEKDVNVGTSGTHTVPRIKAITSKMRMPKSK	*	+	*	+
eu073858	ANDTIDARGTTREMPNKSQGSIQPNLNKEKKRDVNVGTSGTHNVPRIKAITSKMRMPKSK	*	+	*	*
eu161658	GNDTIDAGGSTKKDAKQEQGSIQPNLNKEKEKDVNVGTSGTHTVPRIKAITSKMRMPKSK	NTN[a]	+	*	+
eu182576	GNDTIDAGGSTKKDAKQEQGSIQPNLNKEKEKDVNVGTSGTHTVPRIKAITSKMRMPKSK	N[a]	+	*	+
eu885418	ANDTIDAGGSTKKDAKQEQDSIQPNLNKEKEKDVNVGTSGTHTVPRIKAITSKMRMPKSK	*	+	*	+
fj204164	GNDTIDAGGSTKKDAKQEQGSIQPNLNKEKEKDVNVGTSGTHTVPRIKAITSKMRMPKSK	NTN[b]	+	*	+
fj204165	GNDTIDAGGSTKKDAKQEQGSIQPNLNKEKEKDVNVGTSGTHTVPRIKAITSKMRMPKSK	NTN[b]	+	*	+
fj204166	GNDTIDAGGSTKKDAKQEQGSIQPNLNKEKEKDVNVGTSGTHTVPRIKAITSKMRMPKSK	NTN[b]	+	*	+
fj423032	ANDTIDAGGSTKKDAKQEQGSIQPNLNKEKEKDVNVGTSGTHTVPRIKAITSKMRMPKSK	*	+	*	+
fm201468	ANDTIDAGGSNKKDAKQEQGSIQPNLNKEKDKDVNAGTSGTHTVPRIKAITSKMRMPKSK	*	+	*	+

gq853593	GNDT**IDAGGS**TKKDA**KQEQGSIQP****NLNKEK**EKDVNVGTSGTHTVPRIKAITSKMRMPKSK	N[c]	+	*	+
gq853594	ANDT**IDAGGS**TKKDA**KQEQGSIQP****NLNKEK**EKDVNVGTSGTHTVPRIKAITSKMRMPKSK	N[c]	+	*	+
gq853595	GNDT**IDAGGS**TKKDA**KQEQGSIQP****NLNKEK**EKDVNVGTSGTHTVPRIKAITSKMRMPKSK	N[c]	+	*	+
gq853596	ANDT**IDAGGS**TKKDA**KQEQGSIQP****NLNKEK**EKDVNVGTSGTHTVPRIKAITSKMRMPKSK	N[c]	+	*	+
gq853597	ANDT**IDAGGS**TKKDA**KQEQGSIQP****NLNKEK**EKDVNVGTSGTHTVPRIKAITSKMRMPKSK	N[c]	+	*	+
gq853598	GNDT**IDAGGS**TKKDA**KQEQGSIQP****NLNKEK**EKDVNVGTSGTHTVPRIKAITSKMRMPKSK	N[c]	+	*	+
gq853599	ANDT**IDAGGS**TKKDA**KQEQGSIQP****NLNKEK**EKDVNVGTSGTHTVPRIKAITSKMKMPKSK	N[c]	+	*	+
gq853600	GNDT**IDAGGS**TKKDA**KQEQGSIQP****NLNKEK**EKDVNVGTSGTHTVPRIKAITSKMRMPRSK	N[c]	+	*	+
gq853601	GNDT**IDAGGS**TKKDA**KQEQGSIQP****NLNKEK**EKDVNVGTSGTHTVPRIKAITSKMRMPRSK	N[c]	+	*	+
gq853602	GNDT**IDAGGS**TKKDA**KQEQGSIQP****NLNKEK**EKDVNVGTSGTHTVPRIKAITSKMRMPKSK	N[c]	+	*	+
gq853603	ANDT**IDAGGS**TKKDA**KQEQGSIQP****NLNKEK**EKDVNVGTSGTHTVPRIKAITSKMRMPRSK	N[c]	+	*	+
gq853604	ANDT**IDAGGS**TKKDA**KQEQGSIQP****NLNKEK**EKDVNVGTSGTHTVPRIKAITSKMRMPKSK	NTN[c]	+	*	+
gq853605	ANDT**IDAGGS**TKKDA**KQEQGSIQP****NLNKEK**EKDVNVGTSGTHTVPRIKAITSKMRMPKSK	NTN[c]	+	*	+
gq853606	ANDT**IDAGGS**TKKDA**KQEQGSIQP****NLNKEK**EKDVNVGTSGTHTVPRIKAITSKMRMPKSK	NTN[c]	+	*	+
gq853607	ANDT**IDAGGS**TKKDA**KQEQGSIQP****NLNKEK**EKDVNVGTSGTHTVPRIKAITSKMRMPKSK	NTN[c]	+	*	+
gq853608	ANDT**IDAGGS**TKKDA**KQEQGSIQP****NLNKEK**EKDVNVGTSGTHTVPRIKAITSKMRMPKSK	NTN[c]	+	*	+
gq853609	ANDT**IDAGGS**TKKDA**KQEQGSIQP****NLNKEK**EKDVNVGTSGTHTVPRIKAITSKMRMPKSK	NTN[c]	+	*	+
gq853610	GNDT**IDAGGS**TKKDA**KQEQGSIQP****NLNKEK**EKDVNVGTSGTHTVPRIKAITSKMRMPKSK	NTN[c]	+	*	+
gq853611	ANDT**IDAGGS**TKKDA**KQEQGSIQP****NLNKEK**EKDVNVGTSGTHTVPRIKAITSKMRMPKSK	NTN[c]	+	*	+
gq853612	ANDT**IDAGGS**TKKDA**KQEQGSIQP****NLNKEK**EKDVNVGTSGTHTVPRIKAITSKMRMPKSK	NTN[c]	+	*	+
gq853614	GNDT**IDAGGS**TKKDA**KQEQGSIQP****NLNKEK**EKDVNVGTSGTHTVPRIKAITSKMRMPKSK	NTN[c]	+	*	+
gq853615	ANDT**IDAGGS**TKKDA**KQEQGSIQP****NLNKEK**EKDVNVGTSGTHTVPRIKAITSKMRMPKSK	NTN[c]	+	*	+
gq853616	ANDT**IDAGGS**TKKDA**KQEQGSIQP****NLNKEK**EKDVNVGTSGTHTVPRIKAITSKMRMPKSK	NTN[c]	+	*	+
gq853617	ANDT**IDAGGS**TKKDA**KQEQGSIQP****NLNKEK**EKDVNVGTSGTHTVPRIKAITSKMRMPKSK	NTN[c]	+	*	+
gq853619	ANDT**IDAGGS**TKKDA**KQEQGSIQP****NLNKEK**EKDVNVGTSGTHTVPRIKAITSKMRMPKSK	NTN[c]	+	*	+
gq853620	GNDT**IDAGGS**TKKDA**KQEQGSIQP****NLNKEK**EKDVNVGTSGTHTVPRIKAITSKMRMPKSK	NTN[c]	+	*	+
gq853621	GNDT**IDAGGS**TKRDA**KQEQGSIQP****NLNKEK**EKDVNVGTSGTHTVPRIKAITSKMRMPKSK	NTN[c]	+	*	+
gq853622	ANDT**IDAGGS**TKKDA**KQEQGSIQP****NLNKEK**EKDVNVGTSGTHTVPRIKAITSKMRMPKSK	NTN[c]	+	*	+
gq853623	GNDT**IDAGGS**TKKDA**KQEQGSIQP****NLNKEK**EKDVNVGTSGTHTVPRIKAITSKMRMPKSK	NTN[c]	+	*	+
gq853624	ANDT**IDAGGS**TKKDA**KQEQGSIQP****NLNKEK**EKDVNVGTSGTHTVPRIQAITSKMRMPKSK	NTN[c]	+	*	+
gq853625	ANDT**IDAGGS**TKKDA**KQEQGSIQP****NLNKEK**EKDVNVGTSGTYTVPRIKAITSKMRMPKSK	NTN[c]	+	*	+
gq853627	ANDT**IDAGGS**TKKDA**KQEQGSIQP****NLNKEK**EKDVNVGTSGTHTVPRIKAITSKMRMPKSK	NTN[c]	+	*	+

gq853628	GNDT**IDAGGS**TKKDA**KQEQGSIQPNLNKEK**EKDVNVGTSGTHTVPRIKAITSKMRMPKSK	NTN[c]	+	*	+
gq853629	ANDT**IDAGGS**TKKDA**KQEQGSIQPNLNKEK**EKDVNVGTSGTHTVPRIKAITSKMRMPKSK	NTN[c]	+	*	+
gq853630	ANDT**IDAGGS**TKKDA**KQEQGSIQPNLNKEK**EKDVNVGTSGTHTVPRIKAITSKMRMPKSK	NTN[c]	+	*	+
gq853663	ANDT**IDAGGS**NKKDA**KQEQGSIQPNLNKEK**EKDVNVGTSGTHTVPRIKAITSKMRMPKSK	N[c]	+	*	+
gq853664	GNDT**IDAGGS**NKKDA**KQEQGSIQPNLNKEK**EKDVNVGTSGTHTVPRIKAITSKMRMPKSK	N[c]	+	*	+
gq853665	ANDT**IDAGGS**TKKDA**KQEQGSIQPNLNKEK**EKDVNVGTSGTHTVPRIKAITSKMRMPRSK	N[c]	+	*	+
gq853666	ANDT**IDAGGS**NKKDA**KQEQGSIQPNLNKEK**EKDVNVGTSGTHTVPRIKAITSKMRMPKSK	N[c]	+	*	+
gq853667	GNDT**IDAGGS**TKKDA**KQEQGSIQPNLNKEK**EKDVNVGTSGTHTVPRIKAITSKMRMPRSK	N[c]	+	*	+
gu073999	ANDT**IDAGGS**TKKDA**KQEQGSIQPNLNKEK**EKDVNVGTSGTHTVPRIKAITSKMRMPKSK	*	+	*	+
m95491	GNDT**IDAGGS**TKKDA**KQEQGSIQPNLNKEK**EKDVNVGTSGTHTVPRIKAITSKMRMPKSK	N[c]	+	*	+
u91747	GNDT**IDTGGS**TKKDA**KQEQGSIQPNLNKEK**EKDVNVGTSGTHTVPRIKAITSKMKMPKSK	N[b]	+	*	*
x54636	GNDT**IDAGGS**TKKDA**KQEQVSIQPNLNKEK**EKDVNAGTSGTHTVPRIRAITSKMRMPKSK	N.D.	+	*	+
x68223	GNDT**IDAGGS**TKKDA**KQEQGIIQPNLNKEK**EKDVNVGTSGTHTVPRIKAITSKMRMPKSK	N[b]	+	*	+
x79305	GNDT**IDAGGS**TKKDA**KQEQGSIQPNLNKEK**EKDVNVGTSGTHTVPRIKAITSKMRMPKSK	NTN[b]	+	*	+
x97895	GNDT**IDAGGS**TKKDA**KQEQGSIQPNLNKEK**EKDVNVGTSGTHTVPRIKAITSKMKMPKSK	N[b]	+	*	+

Table S1 (B). The epitope recognized by MAb1129 is shown in green. The epitope recognized by MAb1130 is shown in red. The protein domain shown in pink is highly conserved and different from the epitope recognized by MAb1128. Amino acid substitutions within these regions are shown in black (underlined).

Accession number	N'-terminal residues 1-60		MAb 1128	MAb 1129	MAb 1130
ab185832	ANDT**IDAGGS**SKKDA**RPEQGSIQSNPNKGK**DKDVNAGTSGTHTVPRIKAITSKMRMPKSK	N-Wi[c]	*	+	+
ab295475	ANDT**IDAGGS**SKKDA**RPEQGSIQSNPNKGK**DKDVNAGTSGTHTVPRIKAITSKMRMPTSK	O[b]	*	+	+
ab461451	ANDT**IDAGGS**SKKDA**RPEQGSIQSNPNKGK**DKDVNAGTSGTHTVPRIKAITSKMRMPTSK	N-Wi[c]	*	+	+
ab461452	GNDT**IDAGGS**SKKDA**RPEQGSIQSNPNKGK**DKDVNAGTSGTHTVPRIKAITSKMRMPKSK	N-Wi[c]	*	+	+
ab461453	ANDT**IDAGGS**SKKDA**RPEQGSIQSNPNKGK**DKDVNAGTSGTHTVPRIKAITSKMRMPTSK	N-Wi[c]	*	+	+
af012026	VNET**IDAGGS**SKKDA**RPEQGSIQSNPNKGK**DKDVNVGTSGTHTVPRIKAITSKMRMPQSK	C[b]	*	+	+
af012028	VNET**IDAGGS**SKKDA**RPEQGSIQSNPNKGK**DKDVNVGTSGTHTVPRIKAITSKMRMPQSK	C[b]	*	+	+
aj223595	ANDT**IDAGGS**SKKDA**RPEQGSIQSNPNKGK**DKDVNAGTSGTHTVPRIKAITSKMRMPKSK	*	*	+	+
aj390292	ANDT**IDAGGS**SKKDA**RPEQGSIQSNPNKGR**DKDVNAGTSGTHTVPRIKAITSKMRMPKSK	O[b]	*	+	+
aj390302	VNET**IDAGGS**SKKDA**RPEQGSIQSNPNKGK**DKDVNVGTSGTHTVPRIKAITSKMRMPQSK	O[b]	*	+	+
aj390306	ANDT**IDAGGS**SKKDA**RPEQGSIQSNPNKGK**DKDVNAGTSGTHTVPRIKAITSKMRMPKSK	O[b]	*	+	+
aj584851	ANDT**IDAGGS**SKKDA**RPEQGSIQSNPNKGK**DKDVNAGTSGTHTVPRIKAITSKMRMPKSK	O[b]	*	+	+
aj585196	ANDT**IDAGGS**SKKDA**RPEQGSIQSNPNKGK**DKDVNAGTSGTHTVPRIKAITSKMRMPKSK	O[b]	*	+	+
aj889867	ANDT**IDAGGS**SKKDA**RPEQGSIQSNPNKGK**DKDVNAGTSGTHTVPRIKAITSKMRMPKSK	N-Wi[c]	*	+	+
aj889868	ANDT**IDAGGS**SKKDA**RPEQGSIQSNPNKGK**DKDVNAGTSGTHTVPRIKAITSKMRMPKSK	N-Wi[c]	*	+	+
aj890348	VNET**IDAGGS**SKKDA**RPEQGSIQSNPNKGK**DKDVNVGTSGTHTVPRIKAITSKMRMPQSK	C[b]	*	+	+
aj890350	ANDT**IDAGES**SKKDA**RPEQGSIQSNPNKGK**DKDVNAGTSGTHTVPRIKAITSKMRMPKSK	N-Wi[c]	*	+	*
am113988	ANDT**IDAGGS**SKKDA**RPEQGSIQSNPNKGK**DKDVNAGTSGTHTVPRIKAITSKMRMPKSK	N-Wi[c]	*	+	+
am236790	GNDT**IDAGGS**SKKDA**RPEQGSIQSNPNKGK**DKDVNAGTSGTHTVPRIKAITSKMRMPKSK	O[b]	*	+	+
am236791	VNDT**IDAGGS**SKKDA**RPEQGSIQSNPNKGK**DKDVNAGTSGTHTVPRIKAITSKMRMPKSK	N-Wi[c]	*	+	+
am236793	ANET**IDAGGS**SKKDA**RPEQGSIQSNPNKGK**DKDVNAGTSGTHTVPRIKAITSKMRMPKSK	O[b]	*	+	+
am236794	ANET**IDAGGS**SKKDA**RPEQGSIQSNPNKGK**DKDVNAGTSGTHTVPRIKAITSKMRMPKSK	N-Wi[c]	*	+	+
am236798	ANDT**IDAGGS**SKKDA**RPEQGSIQSNPNKGK**DKDVNAGTSGTHTVPRIKAITSKMRMPKSK	N-Wi[c]	*	+	+
am236806	VNDT**IDAGGS**SKKDA**RPEQGSIQSNPNKGK**DKDVNAGTSGTHTVPRIKAITSKMRMPKSK	N-Wi[c]	*	+	+
am236807	ANDT**IDAGGS**SKKDA**RPEQGSIQSNPNKGK**DKDVNAGTSGTHTVPRIKAITSKMRMPKSK	N-Wi[c]	*	+	+
am236808	GNDT**IDAGGS**SKKDA**RPEQGSIQSNPNKGK**DKDVNAGTSGTHTVPRIKAITSKMRMPKSK	N-Wi[c]	*	+	+

am236809	VNDTIDAGESSKKDARPEQGSIQSNPNKGKDKDVNAGTSGTHTVPRIKAITSKMRMPKSK	N-Wi[c]	*	+	*
am236811	GNDTIDAGGSSKKDARPEQGSIQSNPNKGKDKDVNAGTSGTHTVPRIKAITSKMRMPKSK	N-Wi[c]	*	+	+
am236813	VNDTIDAGGSSKKDARPEQGSIQSNPNKGKDKDVNAGTSGTHTVPRIKAITSKMRMPKSK	O[b]	*	+	+
am236815	GNDTIDAGGSNKKDTRPEQGSIQSNPNKGKDKDVNAGTSGTHTVPRIKAITSKMRMPKSK	N-Wi[c]	*	+	+
am236816	VNDTIDAGGSNKKDARPEQGSIQSNPNKGKDKDVNAGTSGTHTVPRIKAITSKMRMPKSK	N-Wi[c]	*	+	+
am236817	VNDTIDAGGSSKKDARPEQGSIQSNPNKGKDKDVNAGTSGTHTVPRIKAITSKMKMPKSK	N-Wi[c]	*	+	+
am411504	ANDTIDAGGSSKKDARPEQGSIQSNPNKGKDKDVNAGTSGTHTVPRIKAITSKMRMPKSK	*	*	+	+
am931253	ANDTIDAGGSSKKDARPEQGSIQSNPNKGKDKDVNAGTSGTHTVPRIKAITSKMRMPKSK	*	*	+	+
ay742721	ANDTIDAGGSTKKDARPEQGSIQSNPNKGKDKDVNAGTSGTHTVPRIKAITSKMRMPKSK	O[b]	*	+	+
ay742726	ANDTIDAGGSSKKDARPEQGSIQSNPNKGKDKDVNAGTSGTHTVPRIKAITSKMRMPKSK	O[b]	*	+	+
ay742730	ANDTIDAGGSSKKDARPEQGSIQSNPNKGKDKDVNAGTSGTHTVPRIKAITSKMRMPKSK	O[b]	*	+	+
ay742733	ANDTIDAGGSSKKDARPEQGSIQSNPNKGKDKDVNAGTSGTHTVPRIKAITSKMRMPKSK	O[b]	*	+	+
ay745491	ANDTIDAGGSSKKDARPEQGSIQSNPNKGKDKDVNAGTSGTHTVPRIKAITSKMRMPKSK	N-Wi[c]	*	+	+
ay745492	ANDTIDAGGSSKKDARPEQGSIQSNPNKGKDKDVNAGTSGTHTVPRIKAITSKMRMPKSK	N-Wi[c]	*	+	+
ay792597	ANDTMHAGGSSKKDARPEQGSIQSNPNKGKDKDVNAGTSGTHTVPRIKAITSKMRMPKSK	O[a]	*	+	*
dq008213	ANDTIDAGGSSKKDARPEQGSIQSNPNKGKDKDVNAGTSGTHTVPRIKAITSKMRMPKSK	*	*	+	+
dq157178	ANDTIDAGGSSKKDARPEQGSIQSNPNKGKDKDVNAGTSGTHTVPRIKAITSKMRMPKSK	N-Wi[c]	*	+	+
dq157179	ANDTIDAGGSSKKDARPEQGSIQSNPNKGKDKDVNAGTSGTHTVPRIKAITSKMRMPKSK	N-Wi[c]	*	+	+
dq925435	ANDTIDAGGSSKKDARPEQGSIQSNPNKGKDKDVNAGTSGTHTVPRIKAITSKMRMPKGK	*	*	+	+
ef026074	ANDTIDAGGSSKKDARPEQGSIQSNPNKGKDKDVNAGTSGTHTVPRIKAITSKMRMPKSK	O[b]	*	+	+
ef026076	ANDTIDAGGSSKKDARPEQGSIQSNPNKGKDKDVNAGTSGTHTVPRIKAITSKMRMPKSK	O[b]	*	+	+
ef027867	ANDTIDAGGSSKKDARPEQGSIQSNPNKGKDKDVNAGTSGTHTVPRIKAITSKMRMPTSK	*	*	+	+
ef027883	ANDTIDAGGSSKKDARPEQGSIQSNPNKGKDKDVNAGTSGTHTVPRIKAITSKMRMPKSK	*	*	+	+
ef027887	ANDTIDAGGSSKKDARPEQGSIQSNPNKGKDKDVNAGTSGTHTVPRIKAITSKMRMPKSK	*	*	+	+
ef027889	GNDTIDAGGSSKKDARPEQGSIQSNPNKGKDKDVNAGTSGTHTVPRIKAITSKMRMPKSK	*	*	+	+
ef027890	ANDTIDAGGSIKKDARPEQGSIQSNPNKGKDKDVNAGTSGTHTVPRIKAITSKMRMPKSK	*	*	+	+
ef027895	ANDTIDAGGSSKKDARPEQGSIQSNPNKGKDKDVNAGTSGTHTVPRIKAITSKMRMPTSK	*	*	+	+
ef027896	ANDTIDAGGSSKKDARPEQGSIQSNPNKGKDKDVNAGTSGTHTVPRIKAITSKMRMPTSK	*	*	+	+
ef063710	ANDTNDAGGSSKKDARPEQGSIQSNPNKGKDKDVNAGTSGTHTVPRIKAITSKMRMPKSK	*	*	+	-
eu713856	ANDTIDAGGSSKKDARPEQGSIQSNPNKGKDKDVNAGTSGTHTVPRIKAITSKMRMPTSK	N-Wi[a]	*	+	+
eu073859	ANDTIDAGGSSKKDARPEQGSIQSNPNKGKDKDVNAGTSGTHTVPRIKAITSKMRMPKSK	*	*	+	+
fj423031	ANDTIDAGGSSKKDARPEQGSIQSNPNKGKDKDVNAGTSGTHTVPRIKAITSKMRMPKSK	*	*	+	+

gq200836	ANDT**IDAGGS**SKKDA**RPEQGSIQSNPNKGK**DKDVNAGTSGTHTVPRIKAITSKMRMPKSK	N[b]	*	+	+
gq853631	GNDT**IDAGGS**SKKDA**RPEQGSIQSNPNKGK**DKDVNAGTSGTHTVPRIKAITSKMRMPKSK	N-Wi[c]	*	+	+
gq853632	ANDT**IDAGGS**SKKDA**RPEQGSIQSNPNKGK**DKDVNAGTSGTHTVPRIKAITSKMRMPKSK	N-Wi[c]	*	+	+
gq853633	GNDT**IDAGGS**SKKDA**RPEQGSIQSNPNKGK**DKDVNAGTSGTHTVPRIKAITSKMRMPKSK	N-Wi[c]	*	+	+
gq853634	GNDT**IDAGGS**SKKDA**RPEQGSIQSNPNKGK**DKDVNAGTSGTHTVPRIKAITSKMRMPKSK	O[b]	*	+	+
gq853635	ANDT**IDAGGS**SKKDA**RPEQGSIQSNPNKGK**DKDVNAGTSGTHTVPRIKAITSKMRMPKSK	N-Wi[c]	*	+	+
gq853636	GNDT**IDAGGS**SKKDA**RPEQGSIQSNPNKGK**DKDVNAGTSGTHTVPRIKAITSKMRMPKSK	N-Wi[c]	*	+	+
gq853637	GNDT**IDAGGS**SKKDA**RPEQGSIQSNPNKGK**DKDVNAGTSGTHTVPRIKAITSKMRMPKSK	N-Wi[c]	*	+	+
gq853638	ANDT**IDAGGS**SKKDA**RPEQGSIQSNPNKGK**DKDVNAGTSGTHTVPRIKAITSKMRMPKSK	N-Wi[c]	*	+	+
gq853639	GNDT**IDAGGS**SKKDA**RPEQGSIQSNPNKGK**DKDVNAGTSGTHTVPRIKAITSKMRMPKSK	N-Wi[c]	*	+	+
gq853640	ANDT**IDAGGS**SKKDA**RPEQGSIQSNPNKGK**DKDVNAGTSGTHTVPRIKAITSKMRMPKSK	N-Wi[c]	*	+	+
gq853641	GNDT**IDAGGS**SKKDA**RPEQGSIQSNPNKGK**DKDVNAGTSGTHTVPRIKAITSKMRMPKSK	N-Wi[c]	*	+	+
gq853642	GNDT**IDAGGS**SKKDA**RPEQGSIQSNPNKGK**DKDVNAGTSGTHTVPRIKAITSKMRMPKSK	N-Wi[c]	*	+	+
gq853643	ANDT**IDAGGS**SKKDA**RPEQGSIQSNPNKGK**DKDVNAGTSGTHTVPRIKAITSKMRMPKSK	N-Wi[c]	*	+	+
gq853644	GNDT**IDAGGS**SKKDA**RPEQGSIQSNPNKGK**DKDVNAGTSGTHTVPRIKAITSKMRMPKSK	N-Wi[c]	*	+	+
gq853645	ANDT**IDAGGS**SKKDA**RPEQGSIQSNPNKGK**DKDVNAGTSGTHTVPRIKAITSKMRMPKSK	N-Wi[c]	*	+	+
gq853646	ANDT**IDAGGS**SKKDA**RPEQGSIQSNPNKGK**DKDVNAGTSGTHTVPRIKAITSKMRMPKSK	N-Wi[c]	*	+	+
gq853647	GNDT**IDAGGS**SKKDA**RPEQGSIQSNPNKGK**DKDVNAGTSGTHTVPRIKAITSKMRMPKSK	N-Wi[c]	*	+	+
gq853648	ANDT**IDAGGS**SKKDA**RPEQGSIQSNPNKGK**DKDVNAGTSGTHTVPRIKAITSKMRMPTSK	N-Wi[c]	*	+	+
gq853649	ANDT**IDAGGS**SKKDA**RPEQGSIQSNPNKGK**VKDVNAGTSGTHTVPRIKAITSKMRMPKSK	N-Wi[c]	*	+	+
gq853650	ANDT**IDAGGS**SKKDA**RPEQGSIQSNPNKGK**DKDVNAGTSGTHTVPRIKAITSKMRMPKSK	N-Wi[c]	*	+	+
gq853651	ANDT**IDAGGS**SRKDA**RPEQGSIQSNPNKGK**DKDVNAGTSGTHTVPRIKAITSKMRMPKSK	N-Wi[c]	*	+	+
gq853652	ANDT**IDAGGS**SKKDA**RPEQGSIQSNPNKGK**DKDVNAGTSGTHTVPRIKAITSKMRMPKSK	N-Wi[c]	*	+	+
gq853653	GNDT**IDAGGS**SKKDA**RPEQGSIQSNPNKGK**DKDVNAGTSGTHTVPRIKAITSKMRMPTSK	N-Wi[c]	*	+	+
gq853654	ANDT**IDAGGS**SKKDA**RPEQGSIQSNPNKGK**DKDVNAGTSGTHTVPRIKAITSKMRMPKSK	N-Wi[c]	*	+	+
gq853655	ANDT**IDAGGS**SKKDA**RPEQGSIQSNPNKGK**DKDVNAGTSGTHTVPRIKAITSKMRMPKSK	N-Wi[c]	*	+	+
gq853656	GNDT**IDAGGS**SKKDA**RPEQGSIQSNPNKGK**DKDVNAGTSGTHTVPRIKAITSKMRMPKSK	N-Wi[c]	*	+	+
gq853658	GNDT**IDAGGS**SKKDA**RPEQGSIQSNPNKGK**DKDVNAGTSGTHTVPRIKAITSKMRMPKSK	N-Wi[c]	*	+	+
gq853659	ANDT**IDAGGS**NKRDA**RPEQGSIQSNPNKGK**DKDVNAGTSGTHTVPRIKAITSKMRMPKSK	O[b]	*	+	+
gq853661	ANDT**IDAGGS**SKKDA**RPEQGSIQSNPNKGK**DKDVNAGTSGTHTVPRIKAITSKMRMPKSK	N-Wi[c]	*	+	+
gq853662	GNDT**IDAGGS**SKKDA**RPEQGSIQSNPNKGK**DKDVNAGTSGTHTVPRIKAITSKMRMPKSK	N-Wi[c]	*	+	+
gu074000	ANDT**IDAGGS**SKKDA**RPEQGSIQSNPNKGK**DKDVNAGTSGTHTVPRIKAITSKMRMPTSK	*	*	+	+

Table S1(C). Isolates containing significant variation within the epitope recognized by MAb1128.

The MAb1128-like epitopes are shown in blue. The region just before the MAb1128-like epitope is marked in pink. The epitope recognized by MAb1130 is in red. MAb1129-like epitopes are shown in green. Amino acid substitutions within these regions are shown in black (underlined).

Accession number	N'-terminal residues 1-60		MAb 1128	MAb 1129	MAb 1130
ab185831	GNDTIDAGGSTKKDAKQEQGSIQPNLNKGKEKDVNVGTSGTHTVPRIKAITSKMRMPKSK	*	*	*	+
ab185833	GNDTIDAGGSTKKDAKQEQGSIQPNLNKGKEKDVNVGTSGTHTVPRIKAITSKMRMPKSK	N[b]	*	*	+
af255660	GNDTIDTGGSTKKDAKQEQGSIQPKLNKEKEKDVNVGTSGTHTVPRIKAITSKMRMPKSK	O[a]	*	*	*
af325927	GNDTIDAGGSTKKDAKQEQGSIQPILNKEKEKDVNVGTSGTHTVPRIKAITSKMRMPKSK	N[b]	*	*	+
aj223593	ANDTIDAGGNSKKDAKPEQGSIQSNLNKGKDKDVNAGTSGTHTVPRIKAITSKMRMPKSK	*	*	*	*
aj390290	GNDTIDAGGSTKKDAKQEQGSIQPNENKEKEKDVNVGTSGTHTVPRIKAITSKMRMPKSK	N[b]	*	*	+
aj390295	GNDTIDAGGSTKKDAKQEQGSIQPNPNKEKEKDVNVGTSGTHTVPRIKAITSKMRMPKSK	N[b]	*	*	+
aj390296	GNDTIDAGGSTKKDAKQEQGSIQPSLNKEKEKDVNVGTSGTHTVPRIKAITSKMRMPKSK	N[b]	*	*	+
aj390301	ANDTIDAGESSKRDAKPEQGSIQSNLNKGKDKDVNAGTSGTHTVPRIKAITSKMRMPKSK	O[b]	*	*	*
aj390304	GNDTIDAGGSTKKDAKQEQGSIQPNPNKEKEKDVNVGTSGTHTVPRIKAITSKMRMPKSK	N[b]	*	*	+
aj390305	ANDTIDAGGSSKKDARPEQGSIQSNLNKGKDKDVNAGTSGTHTVPRIKAITSKMRMPKSK	O[b]	*	*	+
aj585195	ANDTIDAGGDSKKDAKPEQGSIQSNLNKGKDKDVNAGTSGTHTVPRIKAITSKMRVPKSK	O[b]	*	*	*
am236818	VNDTIDAGGNSKKDAKPEQGSIQSNLNKGKDKDVNAGTSGTHTVPRIKAITSKMRMPKSK	O[b]	*	*	*
am268435	GNDTIDAGGSTKKDAKQEQGSIQPKLNKEKEKDVNVGTSGTHTVPRIKAITSKMRMPKSK	N[c]	*	*	+
ay166866	GNDTIDAGGSTKKDAKQEQGSIQPSLNKEKEKDVNVGTSGTHTVPRIKAITSKMRMPRSK	NTN[b]	*	*	+
ay319647	ANDTIDAGGSSKKDANQEQSSIQPNPNKRKEKDVNVGTSGTHTVPRIKAITSKMRMPKSK	N[c]	*	*	+
ay742714	ANDTIDAGGSTKKDAKQEQSSIQPSLNKEKEKDVNVGTSGTHTVPRIKAITSKMRMPKSK	NTN[b]	*	*	+
ay742715	ANDTIDAGGSNKKDAKQEQSSIQPSLNKEKEKDVNVGTSGTHTVPRIKAITSKMRMPKSK	N[b]	*	*	+
ay742716	ANDTIDAGGSNKKDAKQEQSSIQPSLNKEKEKDVNVGTSGTHTVPRIKAITSKMRMPKSK	N[b]	*	*	+
ay742717	ANDTIDAGGSTKKDAKQEQSSIQPSLNKEKEKDVNVGTSGTHTVPRIKAITSKMRMPKSK	N[b]	*	*	+
ay742718	ANDTIDAGGSNKKDAKQEQGSIQPSLNKEKEKDVNVGTSGTHTVPRIKAITSKMRMPKSK	N[b]	*	*	+
ay742719	ANDTIDAGGSTKKDAKQEQGSIQPSLNKEKEKDVNVGTSGTHTVPRIKAITSKMRMPKSK	N[b]	*	*	+
ay742720	ANDTIDAGGSNKKDAKQEQGSIQPSLNKEKEKDVNVGTSGTHTVPRIKAITSKMRMPKSK	N[b]	*	*	+
ay742722	ANDTIDAGGSTKKDAKQEQSSIQPSLNKEKEKDVNVGTSGTHTVPRIKAITSKMRMPKSK	N[b]	*	*	+

ay742723	ANDTIDAGGSTKKDAKQEQSSIQPSLNKEKEKDVNVGTSGTHTVPRIKAITSKMRMPKSK	N[b]	*	*	+
ay742724	ANDTIDAGGSTKKDAKQEQSSIQPSLNKEKEKDVNVGTSGTHTVPRIKAITSKMRMPKSK	N[b]	*	*	+
ay742727	ANDTIDAGGSNKKDAKQEQGSIQPSLNKEKEKDVNVGTSGTHTVPRIKAITSKMRMPKSK	N[b]	*	*	+
ay742728	ANDTIDAGGSTKKDAKQEQSSIQPSLNKEKEKDVNVGTSGTHTVPRIKAITSKMRMPKSK	N[b]	*	*	+
ay742729	ANDTIDAGGSNKKDAKQEQGSIQPSLNKEKEKDVNVGTSGTHTVPRIKAITSKMRMPKSK	N[b]	*	*	+
ay742731	ANDTIDAGGSNKKDAKQEQGSIQPSLNKEKEKDVNVGTSGTHTVPRIKAITSKMRMPKSK	N[b]	*	*	+
ay742732	ANDTIDAGGSTKKDAKQEQGSIQPSLNKEKEKDVNVGTSGTHTVPRIKAITSKMRMPKSK	N[b]	*	*	+
ay841257	ANDTIDAGGSTKKDAKQEQSSIQPSLNKEKEKDVNVGTSGTHTVPRIKAITSKMRMPKSK	N[b]	*	*	+
ay884982	GNDTIDAGGSTKKDAKQEQGSIQPSLNKEKEKDVNVGTSGTHTVPRIKAITSKMRMPKSK	N[b]	*	*	+
dq217931	ANDTIDAGGNSKKDAKPEQGSIQSNLNKGKDKDVNAGTSGTHTVPRIKAITSKMRMPKSK	*	*	*	*
dq925437	GNDTIDAGGSTKKDAKQEQGSIQPNFNKEKEKDVNVGTSGTYTVPRIKAITSKMRMPKSK	N[c]	*	*	+
ef027866	GNDTIDAGGSTKKDAKQEQGSIQPILNKEKEKDVNVGTSGTHTVPRIKAITSKMRMPKSK	N[c]	*	*	+
ef027884	ANDTIDAGGNSKKDAKPEQGSIQSNLNKGKDKDVNAGTSGTHTVPRIKAITSKMRMPKSK	*	*	*	*
ef027885	ANDTIDAGGSSKKDARPEQGSIQSNLNKGKDKDVNAGTSGTHTVPRIKAITSKMRMPKSK	*	*	*	+
ef027893	GNDTIDAGGSSKKDARPEQGSIQSNLNKGKDKDVNAGTSGTHTVPRIKAITSKMRMPKSK	*	*	*	+
ef027894	GNDTIDAGGSSKKDARPEQGSIQSNLNKGKDKDVNAGTSGTHTVPRIKAITSKMRMPKSK	*	*	*	+
fm200035	ANDTIDAGESNKKDAKPEQGSIQSNLNKGKDKDVNAGTSGTHTVPRIKAITSKMRMPKSK	*	*	*	*
gq853613	GNDTIDAGGSTKKDAKQEQGSIQPNHNKEKEKDVDVGTSGTHTVPRIKAITSKMRMPKSK	*	*	*	+
gq853618	ANDTIDAGGSTKKDAKQEQGSIQPNLNKEEEKDVNVGTSGTHTVPRIKAITSKMRMPESK	*	*	*	+
gq853626	ANDTIDAGGSTKKDAKQEQGSIQPNLSKEKEKDVNVGTSGTHTVPRIKAITSKMRMPKSK	*	*	*	+
gq853660	ANDTIDAGGSSKKDARPEQGSIQSNLNKGKDKDVNAGTSGTHTVPRIKAITSKMRMPKSK	O[b]	*	*	+
s74810	ANDTIDAGGNSKKDAKPEQGSIQSNLNKGNDKDVNAGTSGTHTVPRIKAITSKMRMPKSK	O[b]	*	*	*
s74813	GNDTIDAGGSTKKDAKQEQGSIQPSLNKEKEKDVNVGTSGTHTVPRIKAITSKMRMPKSK	N[b]	*	*	+
u06789	GNDTIDAGGSTKKDAKQEQGIIQPNPNKEKEKDVNVGTSGTHTVPRIKAITSKMRMPTSK	N[b]	*	*	+
u09508	GNDTIDAGGSTKKDAKQEQGSIQPNLIKEKEKDVNVGTSGTHTVPRIKAITSKMRMPKSK	N[b]	*	*	+
x54611	GNDTIDAGGSTKKDAKQEQGSIQPNLNNGKEKDVNVGTSGTHTVPRIKAITSKMRMPKSK	NTN[b]	*	*	+
x92078	GNDTIDAGGSTKKDAKQEQGSIQPILNKEKEKDVNVGTSGTHTVPRIKAITSKMRMPKSK	*	*	*	+
z70237	GNDTIDAGGSTKKDAKQEQGSIQPILNKEKEKDVNVGTSGTHTVPRIKAITSKMRMPKSK	N[a]	*	*	+

Table S1(D). Isolates containing significant variation within the epitope recognized by MAb1129.

The MAb1129-like epitopes are shown in green. The downstream MAB1128-like epitope region is shown in pink. The epitope recognized by MAb1130 is in red. Amino acid substitutions within these regions are shown in black (underlined).

Accession number	N'-terminal residues 1-60		MAb 1128	MAb 1129	MAb 1130
ab256029	GNDTIDAGGSTKKDAKQEQGSIQPNPNKGKEKDVNVGTSGTHTVPRIKAITSKMRMPKSK	N[b]	*	*	+
ab270705	GNDTIDAGGSSKKDARPEQSSIQSNPNKGKDKDVNAGTSGTHTVPRIKAITSKMRMPTSK	N-Wi[c]	*	*	+
ab295476	GNDTIDAGGSTKKDAKQEQGSIQPNPNKGKEKDVNVGTSGTHTVPRIKAITSKMRMPKSK	NTN[a]	*	*	+
af012027	ANDTIDAGGNSKKDAKPEQGSIQPNPNKGKDKDVNAGTSGTHTVPRIKAITSKMRMPKSK	C[b]	*	*	*
af012029	ANDTIDAGVNNKKDAKPEQGSIQPNPNKGKDKDVNAGTSGTHTVPRIKAITSKMRMPKSK	C[b]	*	*	*
af118153	ANDTIDAGGSNKKDAKPEQGSIQSNPNKGKDKDVNAGTFGTHTVPRIKAITPKMRMPKSK	O[a]	*	*	+
af237963	ANDTIDAGGSSKKDAKPEQDSIQPSSNKGKDKDVNAGTSGTHTVPRIKAITSKMRMPKSK	O[b]	*	*	+
af255659	ANDTIDAGGSNKRDAKPEQGSIQSNPNKGKDKDVNAGTSGTHTVPRIKAITSKMRMPKSK	O[b]	*	*	+
af463399	ANDTIDAGESSKKDAKPEQGSIQPKPNKAKDKDVNAGTSGTHAVPRIKAITSKMRMPKSK	N[b]	*	*	*
af522296	ANDTIDAGGSNKKDAKPEQGSIQPNPNKGKDKDVNAGTSGTHTVPRIKAITSKMRMPTSK	*	*	*	+
af525081	ANDTIDAGGSNKRDAKPEQGSIQSNQNKGKDKDVNAGTSGTHTVPRIKAITSKMRMPKSK	*	*	*	+
aj005639	ANDTIDAGENNKKDAKPEQGSIQRNPNKGKEKDVNAGTSGTHTVPRIKAITSKMRMPKSK	C[a]	*	*	*
aj223594	ANDTIDAGGSSKRDAKPEQGSIQSNPNKGKDKDVNAGTSGTHTVPRIKAITSKMRMPKSK	O[a]	*	*	+
aj303093	ANDTIDAGGSSKKDAKPEQGSIQPSPNKGKDKDVNAGTSGTHTVPRIKAITSKMRMPKSK	C[b]	*	*	+
aj303094	ANDTIDAGGSSKKDAKPEQGSIQLNPNKGKDKDVNAGTSGTHTVPRIKAITSKMRMPKSK	C[b]	*	*	+
aj303095	ANDTIDAGESSKKDAKPEQGSIQPNPNKGKDKDVNAGTSGTHTVPRIKAITSKMRMPKSK	C[b]	*	*	*
aj303096	ANDTIDAGEKSKKDAKPEQGSIQRNPNKGEDKDVNAGTSGTHTVPRIKAITSKMRMPKSK	C[b]	*	*	*
aj303097	ANDTIDAGENSKKDAKPEQGSIQRNPNKGKEKDVNAGTSGTHTVPRIKAITSKMRMPKSK	C[b]	*	*	*
aj390297	ADETIDAGENSKKDAKPEQGSIQRNPNKGKDKDVNAGTSGTHTVPRIKAITSKMRMPKSK	O[b]	*	*	*
aj390307	ANETIDAGENSKKDAKPEQGSIQRNPNKGKDKDVNAGTSGTHTVPRIKAITSKMRMPKSK	O[b]	*	*	*
aj439544	ANDTIDAGENSKKDAKPEQGSIQRNPNKGKDKDVNAGTSGTHTVPRIKAITSKMRMPKSK	C[c]	*	*	*
aj439545	ANDTIDAGGSSKKDAKPEQGSIQPTPNKGKDKDVNAGTSGTHTVPRIKAITSKMRMPKSK	C[c]	*	*	+
aj488834	ANDTIDAGGNSKKDAKPEQGSIQPNPNKGKDKDVNAGTSGTHTVPRIKAITSKMRMPKSK	*.	*	*	*
aj890349	ANDTIDAGESSKKDAKPEQGSIQLNPNKGKDKDVNAGTSGTHTVPRIKAITSKMRMPKSK	O[c]	*	*	*

am236795	GNDTIDAGGSSKKDARPEPGSIQSNPNKGKDTDVNAGTSGTHTVPRIKAITSKMRMPNSK	O[b]	*	*	+
am236800	VNDTIDAGGGNKKDAKPEQGSIQPNPNKGKDKDVNAGTFGTHTVPRIKAITSKMRMPKSK	N-Wi[c]	*	*	*
am236801	GNDTIDAGGSNKKDAKPEQGSIQPNPNKGKDKDVNAGTSGTHTVPRIKAITSKMRMPKSK	N-Wi[c]	*	*	+
am236802	ANDTIDAGGSNKKDAKPEQGSIQPNPNKGKDKDVNAGTSGTHTVPRIKAITSKMRMPKSK	N-Wi[c]	*	*	+
am236803	GNDTIDAGGSNKKDAKPEQGSIQPNPNKGKDKDVNAGTSGTHTVPRIKAITSKMRMPKSK	N-Wi[c]	*	*	+
am236804	VNDTIDAGGSNKKDAKPEQGSIQPNPNKGKDKDVNAGTSGTHTVPRIKAITSKMRMPKSK	N-Wi[c]	*	*	+
am236805	GNDTIDAGGSNKKDAKPEQGSIQPNPNKGKDKDVNAGTSGTHTVPRIKAITSKMRMPKSK	N-Wi[c]	*	*	+
am236812	GNDTIDAGGSNKKDAKPEQGSIQSNPNKGKDRDVNAGTSGTHTVPRIKAITSKMRMPKSK	N-Wi[c]	*	*	+
ay061994	ANDTIDAGENSRKDAKPEQGSIQPNPNKGKDKDVNAGTSGTHTVPRIKAITSKMRMPKSK	O[a]	*	*	*
ay512655	ANDTIDAGGSSKKDAKPEQGSIQSNPNKGKDKDVNAGTSGTHTVPRIKAITSKMRMPKSK	*	*	*	+
ay660662	ANDTIDAGGSSKKDAKPEQGSIQPNPNKGKDKDVNAGTSGTHTVPRIKAITSKMRMPKSK	*	*	*	+
ay742725	ANDTIDAGGSTKKDAKPEQGSIQSNPNKGKDKDVNAGTSGTHTVPRIKAITSKMRMPKSK	O[b]	*	*	+
ay841259	ANDTIDAGGSNKKDAKPEQGSIQPNPNKGKDKDVNAGTSGTHTVPRIKAITSKMRMPKSK	O[b]	*	*	+
ay841261	ANDTIDAGGSNKKDAKPEQGSIQPNPNKGKDKDVNAGTSGTHTVPRIKAITSKMRMPKSK	O[b]	*	*	+
ay841262	ANDTIDAGGSNKKDAKPEQGSIQSNPNKGKDKDVNAGTSGTHTVPRIKAITSKMRMPKSK	O[b]	*	*	+
ay841263	ANDTIDAGGSNKKDAKLEQGSIQPNPNKGKDKDVNAGTSGTHTVPRIKAITSKMRMPKSK	O[b]	*	*	+
ay841264	ANDTIDAGGSNKKDAKLEQGSIQPNPNKGKDKDVNAGTSGTHTVPRIKAITSKMRMPKSK	O[b]	*	*	+
ay841265	ANDTIDAGGSNKKDAKPEQGSIQSNPNKGKDKDVNAGTSGTHTVPRIKAITSKMRMPKSK	O[b]	*	*	+
ay841266	ANDTIDAGGSSKKDAKPEQGSIQPNPNKGKDKDVNAGTSGTHTVPRIKAITSKMRMPKSK	O[b]	*	*	+
ay841269	ANDTIDAGGSSKKDAKPEQGSIQPNPNKGKDKDVNAGTSGTHTVPRIKAITSKMRMPKSK	O[b]	*	*	+
ay884985	ANDTIDTGGSSKKDAKPEQGSIQSNPNKGKDKDVNAGTSGTHTVPRIKAITSKMRMPKSK	O[b]	*	*	*
d12539	ANDTIDAGGGNKRDAKLEQDSIQSNPNKGKDKDVNAGTSGTHTVPRIKAITSKMRMPKSK	O[b]	*	+	*
dq309028	ANDTIDAGESSKKDAKPEQGSIQPKPNKGKDKDVNAGTSGTHAVPRIKAITSKMRMPKSK	*	*	*	*
dq925436	ANDPIDAGENNKKDAKPAQGSIQPSPNKGKDKDVNAGTSGTHTVPRIKAITSKMRMPKSK	C[c]	*	*	*
ef027888	ANDTIDAGGSNKRDAKPEQSSIQSNPNKGKDKDVNAGTSGTHTVPRIKAITSKMRMPKSK	*	*	*	+
ef027900	GNDTIDAGGSSKKDARLEQGSIQSNPNKGKDKDVNAGTSGTHTVPRIKAITSKMRMPKSK	*	*	*	+
ef455803	ANDTIDAGGSSKKDAKPEQGSIQPNPSKGKDKDVNAGTSGTHTVPRIKAITSKMRMPKSK	*	*	*	+
ef558545	ANDTIDAGESSKKDAKPEQGSIQLNPNKGKDKDVNAGTSGTHTVPRIKAITSKMRMPKSK	N-Wi[a]	*	*	*
eu252529	ANDTIDAGGISKKDAKPEQGSIQPSPNKGKDKDVNAGTSGTHTVPRIKAITSKMRMPKSK	C[b]	*	*	*
eu482153	ANDTIDAGGSSKRDAKPEQSSIQSNPNKGKDKDVNAGTSGTHTVPRIKAITSKMRMPKSK	*	*	*	+
fj214726	ANDTIDAGGSSKKDAKPEQGSIQPNPNKGKDKDVNVGTSGTHTVPRIKAITSKMRMPTSK	*	*	*	+
fm244834	ANDTIDAGENNKKDAKPAQGSIQPNSTKGKDKDVNAGTSGTHTVPRIKAITSKMRMPKSK	*	*	*	*

gq853657	GNDTIDAGGSSKKDARPEQGSIQSSPNKGKDKDVNAGTSGTHTVPRIKAITSKMRMPKSK	*	*	*	+
m11598	ANDTIDTGGNSKKDVKPEQGSIQPSSNKGKEKDVNAGTSGTHTVPRIKAITAKMRMPKSK	C[b]	*	*	*
m81435	ANDTIDAGGNNKKDVKPEQGSIQLNPNKGKDKDVNAGTSGTHTVPRIKAITSKMRMPKSK	*	*	*	*
u09509	ANDTIDAGGSNKKDTKPEQSSIQSNPNKGKDKDVNAGTSGTHTVPRIKAITSKMRMPKSK	O[b]	*	*	+
u10378	ANDTIDAGGSSKKDAKPEQDSIQPSSNKGKDKDVNAGTSGTHTVPRIKAITSKMRMPKSK	C[b]	*	*	+
u25672	ANDTIDAVESNKKESKPEQGSIQSNSNKGIDKDVNAGTSGTHTVPRIKAITSKMRMPKSK	*	*	*	*
x12456	ANDTIDAGGSNKKDAKPEQGSIQPNPNKGKDKDVNAGTSGTHTVPRIKAITSKMRMPTSK	O[b]	*	*	+
x14136	ANDTIDAGGNNKKDAKPEQSSIQSNLSKGKDKDVNVGTSGTHTVPRIKAITSKMRMPRSK	O[b]	*	*	*
x54058	ANDTIDAVGDNKKDAKPEQGSIQSNPNKGKEKDVNAGTSGTHTVPRIKAITPKMRMPKSK	*	*	*	*
x68221	ANDTIDAGGSSKKDTKPEQGSIQPNPNKGKDKDVNVGTSGTHTVPRIKAITSKMRMPTSK	N[b]	*	*	+
x68222	ANDTINAGGSNKKDTKPEQSSIQSNLIKGKDKDVNAGTSGTHTVPRIKAITSKMRMPKSK	O[b]	*	*	*
x68224	ANDKIDAGENSKKDAKPEQGSIQPSSNKGKEKDVNAGTSGTHTVPRIKAITAKIRMPKSK	N[b]	*	*	*
x68225	ANDTIDAGENSKKDAKPEQGSIQPKPNKVKDKDVNAGTSGTHAVPRIKAITSKMRMPKSK	O[b]	*	*	*
x68226	GNDTIDAGGNNKKDVKPEQGSIQLNPNKGKDKDVNAGTSGTHTVPRIKAITSKMRMPKSK	O[b]	*	*	*
z70238	ANDTIDAGESSKKDAKPEQGSIQLNPNKGKDKDVNAGTSGTHTVPRIKAITSKMRMPKSK	O[b]	*	*	*
z70239	ANDTIDAGESSKKDVKPEQGSIQLNPNKGKDKDVNAGTSGTHTVPRIKAITSKMRMPKSK	O[b]	*	*	*
